# Genetic analysis of 19 X chromosome STR loci for forensic purposes in four Chinese ethnic groups

**DOI:** 10.1038/srep42782

**Published:** 2017-02-17

**Authors:** Xingyi Yang, Xiaofang Zhang, Junyong Zhu, Linli Chen, Changhui Liu, Xingling Feng, Ling Chen, Huijun Wang, Chao Liu

**Affiliations:** 1 Department of Forensic Medicine, School of Basic Medical Sciences, Southern Medical University, Guangzhou, Guangdong Province 510515, P.R. China; 2 Guangzhou Forensic Science Institute, Guangdong Province Key Laboratory of Forensic Genetics, Guangzhou 510030, P.R. China; 3 AGCU ScienTech Incorporation, Wuxi 214174, P.R. China

## Abstract

A new 19 X- short tandem repeat (STR) multiplex PCR system has recently been developed, though its applicability in forensic studies has not been thoroughly assessed. In this study, 932 unrelated individuals from four Chinese ethnic groups (Han, Tibet, Uighur and Hui) were successfully genotyped using this new multiplex PCR system. Our results showed significant linkage disequilibrium between markers DXS10103 and DXS10101 in all four ethnic groups; markers DXS10159 and DXS10162, DXS6809 and DXS6789, and HPRTB and DXS10101 in Tibetan populations; and markers DXS10074 and DXS10075 in Uighur populations. The combined powers of discrimination in males and females were calculated according to haplotype frequencies from allele distributions rather than haplotype counts in the relevant population and were high in four ethnic groups. The cumulative powers of discrimination of the tested X-STR loci were 1.000000000000000 and 0.999999999997940 in females and males, respectively. All 19 X-STR loci are highly polymorphic. The highest Reynolds genetic distances were observed for the Tibet-Uighur pairwise comparisons. This study represents an extensive report on X-STR marker variation in minor Chinese populations and a comprehensive analysis of the diversity of these 19 X STR markers in four Chinese ethnic groups.

Autosomal STR markers are well-established and highly effective tools widely used for genetic identity and relationship testing^1^. X chromosome STRs, a complementary tool to autosomal STR and mitochondrial DNA (mtDNA) markers, can be used in forensic investigations such as complex kinship analysis^2^. For example, X-STR loci are especially useful for half-sister deficiency paternity cases^3^,^4^. Moreover, higher mean exclusion chance (MEC) values are obtained when using X chromosome markers in trios involving daughters^4^.

The use of X-STRs requires a precise knowledge of not only allele and haplotype frequencies but also the genetic linkage and linkage disequilibrium (LDE) status among markers^5^. Linkage refers to the co-segregation of closely located loci in a pedigree, while LDE measures allele co-segregation at a population level^6^. In our unpublished data obtained from Southern Han family samples, the analyzed 19 X-STR loci multiplex system included seven clusters of closely linked markers: DXS10148-DXS10135-DXS8378, DXS10159-DXS10162-DXS10164, DXS7132-DXS10079-DXS10074-DXS10075, DXS6809-DXS6789, DXS7424-DXS101, DXS10103-HPRTB-DXS10101 and DXS10134-DXS7423 (located at Xp22, the centromere, Xq12, Xq21, Xq22, Xq26, and Xq28, respectively and each spanning less than 3 cM, similar to the previous research^5^) which increasing the power of discrimination for joint consideration of many X STRs at a time. LDE can be assessed from allele and haplotype frequencies and alleles of closely linked X chromosomal loci can be evaluated as a haplotype rather than single STRs. However, grouping markers into haplotypes may lead to partially redundant information (corresponding to reduce the markers used in multiplex system) when performing kinship testing^7^. Therefore, it is necessary to investigate the LDE of the 19 above-mentioned markers and to calculate the efficacy of these loci through single locus and haplotype frequency analyses to assess their potential use in forensic practices.

## Results and Discussion

### Polymorphism

The genotyping results of the 932 unrelated individuals from the four ethnic groups were successfully typed with the newly developed 19 X-STR loci multiplex system. Allele frequencies between female and male samples in all ethnic groups were not significantly different in the examined loci based on a Wilcoxon signed-ranks test (p ≤ 0.05). Hardy-Weinberg equilibrium (HWE) tests were performed on female samples. Based on a significance level of 0.05, the DXS10079 and DXS7424 markers in the Southern Han population; DXS10135 and DXS10134 in the Tibetan population; DXS10148, DXS10159 and DXS101 in the Uighur population; and DXS6809 in the Hui population all showed departures from HWE. However, no significant deviations from HWE were observed after Bonferroni corrections (P = 0.05/171 = 0.00029).

For these 932 samples, the number of observed alleles varies from 8 to 32 across the different loci. The allele frequencies are shown in Supplementary Tables S1–S10 and the power of discrimination in those females (PD_f_) and males (PD_m_), the polymorphism information content (PIC), the observed heterozygosity (Ho), the expected heterozygosity (He), the mean exclusion chance (MEC), the combined power of discrimination for the females (CDP_f_) and males (CDP_m_), and the combined mean exclusion chance in duo cases (CMEC_d_) for the 19 loci in the Southern Han, Tibetan, Uighur and Hui ethnic groups were all shown in Tables 1, 2, 3, 4, 5, 6, 7, 8, 9 and 10. The typing results for the 9947A control DNA were consistent with those reported in the X chromosome database shown in Supplementary Tables S1–S10. Ho and He are both greater than 0.7 for all markers and, specifically, greater than 0.75 for the DXS8378, DXS10162, DXS10164, DXS7424, DXS7423, DXS10148, DXS10135, DXS10159, DXS10101 and DXS10134 markers. The PIC values of all the selected loci were greater than 0.6 except for those of the DXS8378 marker in the Southern Han and Hui populations, the DXS10164 marker in all groups, and the DXS7423 marker in the Southern Han, Tibetan and Hui populations. The finding of low PIC value in DXS7423 was consistent to the result in Guanzhong Han, Shaanxi province, Western China^8^. The PIC values for the DXS10134, DXS10135, DXS10148 and DXS10101 markers were all greater than 0.8 across all ethnic groups. Meanwhile, the PIC values for the DXS10164 and DXS7423 markers were less than 0.5, which is consistent with the results of Liu *et al*.^9^. We found that DXS10134, DXS10079, DXS10135, and DXS10101 were the most polymorphic loci. All markers possessed high forensic efficiency values within the studied population samples, supporting the benefits of using multiplexes in forensic practices.

### Linkage disequilibrium

A previous study showed that LDE between markers more than 5 Mb apart is unlikely^10^. To validate this theory, LDE was estimated for all pairs of markers in the four population groups. In addition, gametic associations were tested for all pairs of loci in the male samples^11^. The P values for the LDE exact tests are listed in Table 11. Significant associations were found between all pairs, including between DXS10103 and DXS10101 in all four ethnic groups; between DXS10159 and DXS10162, DXS6809 and DXS6789, HPRTB and DXS10101 in the Tibetan population; and between DXS10074 and DXS10075 in the Uighur population. These pairs showed a significant LDE even after Bonferroni correction (P = 0.05/171 = 0.00029). These results suggested that these loci pairs could be treated as haplotype clusters or blocks. For markers showing strong LDE, population data could directly lead to the estimation of haplotype frequencies. The haplotype frequencies and the forensic parameters for DXS10103-DXS10101 in all four ethnic groups; for DXS10159-DXS10162, DXS6809-DXS6789, and DXS10103-HPRTB-DXS10101 in the Tibetan population; and for DXS10074 –DXS10075 in the Uighur population are shown in Supplementary Tables S11–S15. Seventy-five haplotypes were observed for the DXS10103-DXS10101 pair in all 631 male samples, and the PIC and PD_m_ values for this haplotype were both greater than 0.9. The DXS10103-DXS10101 pair was had also been treated as haplotype in Shanghai Han and Taiwanese Han populations in previous studies^12^,^13^.

**Figure 1:**
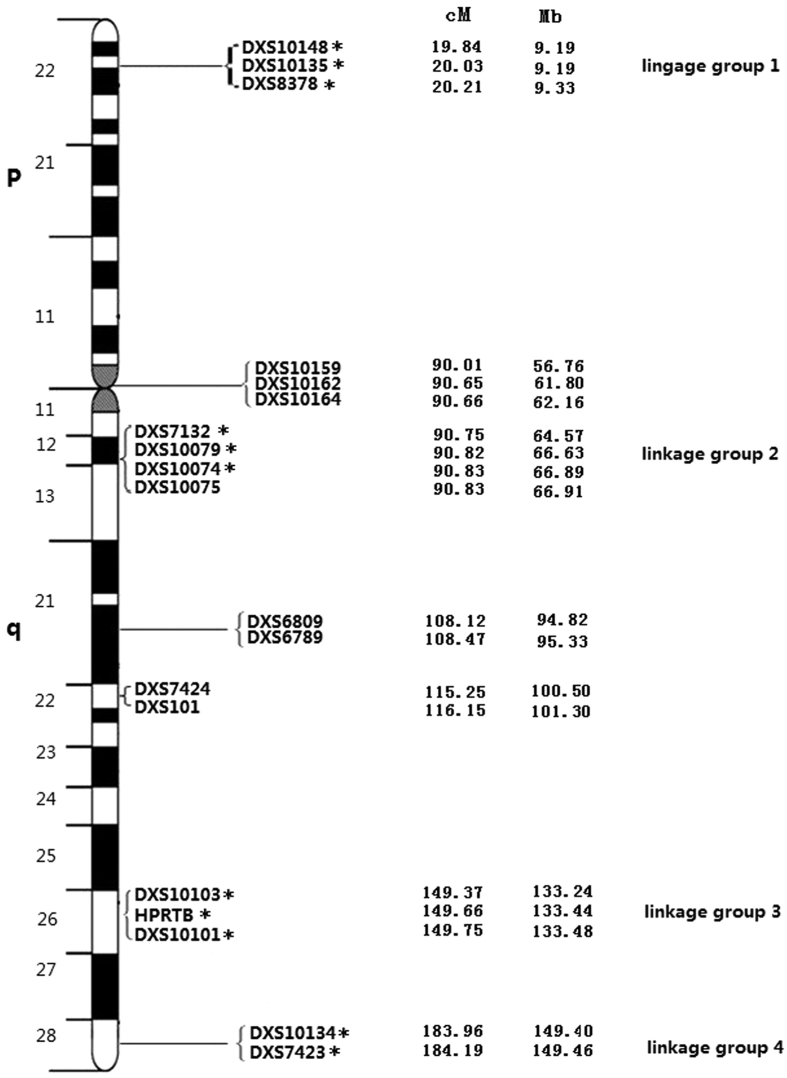
The ideogram of the X-chromosome describes the genetic positions of the 19 X-STR loci and their physical location in the X chromosome. Distances from the p-telomere are shown in cM and Mb. Asterisks (*) indicate the 11 X-STR loci that are shared with the Investigator Argus X-12 kit (Qiagen, Hilden, Germany).

There are 11 X-STR loci that are also used for genetic testing in the Investigator Argus X-12 human identification kit (Qiagen, Hilden, Germany)^12^. These 11 shared loci were marked with an asterisk in Fig. 1. According to previous studies, even when the physical distance between loci is very small, recombination and crossing-over might still happen^14^. While DXS101-DXS7424 and DXS6789-DXS7424 were previously reported to be in linkage disequilibrium in a northwestern Italian population and other populations^15^,^16^, no evidence for LDE in DXS101-DXS7424 was observed in this study. Further studies should be performed to more thoroughly assess the linkage between markers and better define the proposed linkage groups.

The forensic statistical parameters found for the five haplogroups are shown in Table 12. PIC values of all loci were greater than 0.95 except for DXS10159-DXS10162 in the Tibetan population and DXS10074-DXS10075 in the Uighur population. The He values are all greater than 0.95, and the haplotype diversity values are greater than 0.95 except for DXS6809-DXS6789 and DXS10103-HPRTB-DXS10101 in the Tibetan population and for DXS10103-DXS10101 in the Hui population. The PD_f_ values are all greater than 0.99, and the MEC_d_ values are all greater than 0.9 except for DXS10159-DXS10162 in the Tibetan population. All haplotypes showed high forensic efficiency values that reflect their utility for forensic uses.

### Comparisons among the four ethnic groups

Allele frequency distribution comparisons were performed among these four ethnic populations. The allele frequency distribution showed significant differences for most of the loci among these four Chinese ethnic groups; based on these results, population analyses were performed separately for each individual population (Supplementary Table S16). Significant differences were found for 11 loci between the Han and Tibetan populations, for 1 locus between the Han and Hui populations, and for 16 loci between the Han and Uighur populations. Based on these results, the Hui population is genetically closer to the Southern Han populations than to the Tibetan and Uighur populations.

The allele frequencies of these four Chinese populations were also compared with those from other populations, including the Chinese Northern Han population^17^, a Korean population^18^, a population from Japan^19^, a population from northern Germany^20^, the Polish Tatars^21^, a northern Italian population^22^, a population from Spain^23^, and an Ecuadorian Kichwa population^24^ (Tables S17–S20). We found no significant differences between the Southern Han and Northern Han populations. This result was not consistent with Shin’s findings^25^, probably because of the different loci assayed. Meantime, the allele frequency distribution comparisons between Southern Han and Guanzhong Han,which study concerning the same panel as our^8^, presented no significant differences in Table S22. While the value are much greater among Guanzhong Han and Tibet. Uighur. Hui than Southern Han ethnic groups in PIC, He, CDP_f_, CDP_m_ CMEC_t_ and CMEC_d_^8^ in Table S23. We did find significant differences for most of the loci among the Southern Han, Tibetan, Uighur, Japanese, Northern German, Polish Tatars, Northern Italian, Spanish and Ecuadorian Kichwa populations (Supplementary Tables S17–S20). However, we found no significant differences among the Southern Han, Hui and Korean populations, except for the DXS8378 and DXS6789 loci.

The F-statistic (Fst) is often used in forensic sciences to measure population substructure^23^. The maximum observed Fst value was 0.01142 (p = 0.00000 ± 0.0000) for the Tibetan and Uighur populations, whereas the minimum Fst value was 0.00128 (p = 0.46847 ± 0.0572) for the Southern Han and Hui populations (Table 13). These results were consistent with the existence of population substructure within the above mentioned populations. However, these results differ from previous STR studies that showed the smallest and the largest genetic distance between the Southern Han and Uighur populations and the Tibetan and Hui populations respectively^26^. A possible explanation for this discrepancy might be that the Hui populations assayed in the two studies are from different geographical regions in China (Kansu and Sinkiang in a previous study and Ningxia Hui Autonomous region in our study).

### Forensic efficiency parameter data

The forensic efficiency parameter data were calculated based on the observed haplotype frequencies when loci were in LDE and allele frequencies in the four ethnic groups, respectively. Therefore, each haplotype is supposed to behave as an allele. The 19 markers are treated as 18 loci in the Southern Han population, as 15 loci in the Tibetan population, as 17 loci in the Uighur population and as 18 loci in the Hui population. The CDPf value was 1.000000000000000, the CDPm value was over 0.999999999997940, the CMECd value was above 0.999999991939326, and the CMECt value was above 0.999999999989069 (Table 14). The CDP and CMEC values were in declining when LDE loci was treated as haplotype rather than just separated. Contributed to this theory, the values of CDP_m_ and CMEC shown smaller in our Southern Han study than in Guanzhong Han which calculated the forensic statistical parameters on allele frequencies^8^. These results showed that the 19 X-STR loci were highly polymorphic and could provide valuable information for forensic analysis^13^. This set of markers may indeed be very useful for kinship testing, as well as for human identification.

### A recombination study of two-generation families with two or more children

Pairwise linkage studies and recombination fraction (θ) calculations were performed for the 19 X-STR loci. The maximum likelihood (LOD) scores for all pairwise linkage analyses in females are shown in the Supplementary Table S21. Several marker pairs showed significant linkage (maximum LOD scores >3). The number of informative meioses ranged from 48 to 87. LOD scores and recombination fractions for adjacent X-STR markers are listed in Table 15. The recombination fraction estimation is necessary for the calculation of likelihood ratios when linked markers are used. It has been previously shown that X-STR recombination rates among populations may differ^27^,^28^. In our study, recombination among the STR clusters was inferred from Southern Han families with two or more children. We did not observe many recombination events between tightly linked markers, though they had been previously found by other researchers between the DXS10079-DXS10074 and the DXS6809-DXS6789 markers with physical distances <1.0 Mb^29^. As suggested by previous reports, recombination estimates should be taken with caution when closely linked X-STRs are considered as stable haplotypes in kinship analysis^30^. However, no recombination events were observed within the seven linked clusters in our study. In our study, the recombination fractions observed for all pairs are in the 95% CIs. More family samples and/or more generation pedigrees are needed to obtain a better estimation of recombination events.

### Phylogenetic analyses

**Figure 2:**
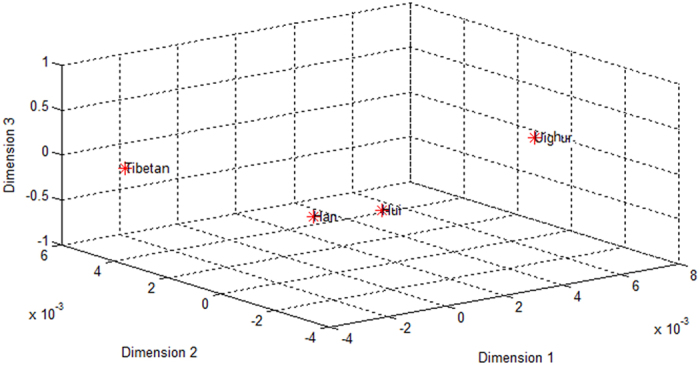
3-D multidimensional scaling (MDS) plot of the four populations (Han, Tibetan, Uighur and Hui) built using Matlab and based on the Reynolds genetic distances. Han short for Southern Han.

As shown in Table 16, the Reynolds study findings showed that the smallest genetic distance between the Southern Han and the Hui populations (0.00128) followed by the Southern Han and the Tibetan populations (0.00631) and the Tibetan and Hui populations (0.00722). As to the largest genetic distance, first one was between the Tibetan and Uighur populations (0.01149), followed by the Han and Uighur populations (0.01075) and the Hui and Uighur populations (0.00900). Based on the Reynolds study, multidimensional scaling (MDS) analysis was performed to evaluate the phylogenetic relationships among the four Chinese ethics groups (Fig. 2) (the significance of the MDS plot data was confirmed using a chi-square test). The Tibetan and Uighur populations at the upper portions of MDS plot segregated as distant outliers, revealing that the Hui and Han population were more genotypic resembling, which may due to their geographical proximity and historic distributions. A possible explanation is that intra-population marriages are more frequent in Han and Hui populations, while inter-population marriages are more common in Tibetan and Uighur populations.

## Conclusions

In this study, we investigated genetic polymorphisms in four Chinese ethnic groups. We tested linkage disequilibrium in 19 X-STR loci and found that these X-STR loci were not independent from each other. Haplotypes of loci in LDE was crucial and meaningful to calculate the exact value of CDP and CMEC in relationship identification case and kinship testing. Hence, allele and haplotype frequencies were both considered when we calculated forensic parameters in this study. In addition, the results indicated that most X-STR allele frequency were shown in a specific population. What is more, the different STR loci applied in genectic distanct calculation contribute to the estimation of far or close relationship among the ethnic groups. Moreover, to achieve a better understanding of genetic structure and inter-population relationships, larger sample sizes from wider geographic area are needed for further evaluation.

## Materials and methods

### Sample collection and DNA extraction

In this study, we collected blood from 932 individuals with no relationship from four ethnic groups in Mainland China with informed consent. Han is the main ethnic group in China, while Tibetan, Uighur and Hui populations are minorities. Our sample included 308 Han subjects (106 females and 202 males) from the Guangdong, Jiangxi, Hunan, and Guangxi Zhuang Autonomous Region in Southern China; 213 Tibetan subjects (61 females and 152 males) from Lhasa City in Tibet Autonomous Region; 211 Uighur subjects (66 females and 145 males) from Korla City in Xinjiang; and 200 Hui subjects (68 females and 132 males) from the Ningxia Hui Autonomous region. Additionally, 40 two-generation Southern Han families with two or more children (94) were tested for the recombination study. AmpFlSTR Identifiler PCR kit purchased from Applied Biosystems, were utilized. Each potential blood donor was investigated for their aboriginal ancestry before and after sample collecting. Only unrelated individuals were sampled. Human blood samples were collected upon approval by the Ethics Committee at the Institute of Forensic Sciences, Ministry of Justice, P R China. All the methods were carried out in accordance with the approved guidelines of the Institute of Forensic Sciences, Ministry of Justice, PR China.

We extracted DNA from samples with magnetic beads (DNA IQ System) on the Maxwell 16 Research System (Promega, Madison WI, USA) and made quantification analysis by 7500 Real-time PCR System following the Human DNA Quantification Kit instruction manual (Thermo Fisher Scientific). Co-amplification of 19 X-STR loci (DXS7423, DXS10148, DXS10159, DXS6809, DXS7424, DXS8378, DXS10164, DXS10162, DXS7132, DXS10079, DXS6789, DXS101, DXS10103, DXS10101, HPRTB, DXS10075, DXS10074, DXS10135 and DXS10134) was performed by following the protocol described in the validation research^31^. For PCR experiment, 1 μL of template DNA, 4 μL of reaction mix, 2 μL of primers, 0.2 μL of A-Taq DNA polymerase, and sdH_2_O were added to a volume of 10 μL solution for reaction. The same cycling parameters were selected for the direct amplification of our samples^31^, with a 1.2 mm punch from FTA blood cards.

### Markers and genotyping

The amplified products were resolved and detected by capillary electrophoresis (CE) with PO denaturing polymers (Thermo Fisher Scientific) in the AB 3130xl Genetic Analyzer (Applied Biosystems, Foster City, CA) following the manufacturer’s manual. The 9947A cell line (Promega, Madison WI, USA) was used as a positive control in all experiments. Negative controls were also included in all experiments. The CE conditions were as follows: sample injection for 5 s at 3 kV, electrophoresis at 15 kV for 1500 s at 60 °C. Gene fragment sizes were determined with GeneMapper ID software (v.3.5) at the detection threshold of 50 RFU.

### Analytical method

The allele and haplotype frequencies for the 19 X-STR were calculated using PowerStat version 1.2 (Promega, Madison WI, USA)^32^. For the male samples^33^, pairwise LD between all pairs of the 19 loci and HWE were tested for each locus using Powermarker software (version 3.25)^34^. For the female samples, Fst and Reynolds genetic distances were calculated using ARLEQUIN software(version 3.5)^35^. MATLAB software (version R2013a) was conducted to obtain forensic parameters based on following allele and haplotype frequencies: Ho, He, PIC^36^, PD_f_, PD_m_. While MEC were measured by referring to methods proposed by Desmarais *et al*.^37^, while CDP_f_, CDP_m_, CMEC_d_, CMEC_t_ and the MDS plot were calculated according to Zhang *et al*.^13^. The maximum LOD scores and θ were estimated using the Mendel v12 software based on the LOD method described in ref. 38. Then, 95% CIs for θ were computed using this online tool http://statpages.org/confint.html. Allele and haplotype frequency distributions for the four ethnic groups were compared with a Chi-square test using SPSS 16.0 with 10,000 permutations^39^.

## Additional Information

**How to cite this article**: Yang, X. *et al*. Genetic analysis of 19 X chromosome STR loci for forensic purposes in four Chinese ethnic groups. *Sci. Rep.* **7**, 42782; doi: 10.1038/srep42782 (2017).

**Publisher's note:** Springer Nature remains neutral with regard to jurisdictional claims in published maps and institutional affiliations.

## Supplementary Material

Supplementary TableS 1-22

## Figures and Tables

**Table 1:** Forensic parameters of 19 X-STR loci among the four ethnic populations.

	DXS10159				DXS6809			
	Han	Tibet	Uighur	Hui	Han	Tibet	Uighur	Hui
PIC	0.7424	0.7621	0.7452	0.7400	0.7744	0.7536	0.7659	0.7735
PD_f_	0.9154	0.9261	0.9188	0.9142	0.9336	0.9217	0.9288	0.9325
PD_m_	0.7774	0.7932	0.7763	0.7754	0.8014	0.7861	0.7950	0.8016
Ho	0.8580	0.8520	0.7580	0.7500	0.7540	0.6890	0.8480	0.7790
He	0.8481	0.8653	0.8469	0.8459	0.8586	0.8423	0.8518	0.8589
MEC_t_	0.7424	0.7621	0.7452	0.7400	0.7744	0.7536	0.7659	0.7735
MEC_d_	0.6108	0.6345	0.6147	0.6078	0.6505	0.6239	0.6400	0.6489

**Table 2:** Forensic parameters of 19 X-STR loci among the four ethnic populations.

	DXS10134				DXS10074			
	Han	Tibet	Uighur	Hui	Han	Tibet	Uighur	Hui
PIC	0.8487	0.8200	0.8614	0.8433	0.7207	0.7728	0.7679	0.7441
PD_f_	0.9668	0.9555	0.9716	0.9647	0.9035	0.9325	0.9305	0.9165
PD_m_	0.8631	0.8383	0.8738	0.8586	0.7592	0.8006	0.7956	0.7786
Ho	0.7670	0.8220	0.8480	0.8380	0.7340	0.6560	0.7880	0.7210
He	0.8919	0.8663	0.9030	0.8872	0.8098	0.8540	0.8486	0.8305
MEC_t_	0.8487	0.8200	0.8614	0.8433	0.7207	0.7728	0.7679	0.7441
MEC_d_	0.7496	0.7106	0.7679	0.7420	0.5852	0.6488	0.6427	0.6128

**Table 3:** Forensic parameters of 19 X-STR loci among the four ethnic populations.

	DXS10079				DXS10162			
	Han	Tibet	Uighur	Hui	Han	Tibet	Uighur	Hui
PIC	0.7908	0.7562	0.7790	0.7899	0.7291	0.6682	0.7358	0.7337
PD_f_	0.9414	0.9235	0.9361	0.9410	0.9090	0.8711	0.9129	0.9117
PD_m_	0.8152	0.7876	0.8048	0.8145	0.7647	0.7171	0.7700	0.7683
Ho	0.7480	0.7000	0.7420	0.8240	0.7480	0.8030	0.7120	0.6760
He	0.8893	0.8591	0.8780	0.8885	0.8497	0.7967	0.8556	0.8537
MEC_t_	0.7908	0.7562	0.7790	0.7899	0.7291	0.6682	0.7358	0.7337
MEC_d_	0.6709	0.6278	0.6564	0.6703	0.5952	0.5255	0.6030	0.6006

**Table 4:** Forensic parameters of 19 X-STR loci among the four ethnic populations.

Allele	DXS6789				DXS10075			
	Han	Tibet	Uighur	Hui	Han	Tibet	Uighur	Hui
PIC	0.7561	0.7846	0.7831	0.7736	0.6677	0.6389	0.6710	0.6565
PD_f_	0.9248	0.9380	0.9373	0.9329	0.8713	0.8534	0.8738	0.8626
PD_m_	0.7852	0.8108	0.8094	0.8012	0.7154	0.6882	0.7172	0.7094
Ho	0.7741	0.7541	0.7273	0.8676	0.7240	0.6560	0.7420	0.6320
He	0.8637	0.8919	0.8903	0.8813	0.7805	0.7508	0.7824	0.7739
MEC_t_	0.7561	0.7846	0.7831	0.7736	0.6677	0.6389	0.6710	0.6565
MEC_d_	0.6281	0.6626	0.6613	0.6491	0.5253	0.4938	0.5297	0.5129

**Table 5:** Forensic parameters of 19 X-STR loci among the four ethnic populations.

	DXS7132				DXS7423			
	Han	Tibet	Uighur	Hui	Han	Tibet	Uighur	Hui
PIC	0.7026	0.6738	0.6973	0.6946	0.4295	0.4348	0.6135	0.4326
PD_f_	0.8937	0.8785	0.8892	0.8877	0.6791	0.6836	0.8356	0.6823
PD_m_	0.7427	0.7128	0.7412	0.7385	0.5198	0.5351	0.6668	0.5153
Ho	0.7280	0.6070	0.5910	0.6470	0.6480	0.5570	0.6360	0.4850
He	0.8488	0.8146	0.8470	0.8440	0.5940	0.6116	0.7620	0.5889
MEC_t_	0.7026	0.6738	0.6973	0.6946	0.4295	0.4348	0.6135	0.4326
MEC_d_	0.5643	0.5316	0.5580	0.5548	0.2937	0.3000	0.4667	0.2956

**Table 6:** Forensic parameters of 19 X-STR loci among the four ethnic populations.

	DXS7424				DXS10164			
	Han	Tibet	Uighur	Hui	Han	Tibet	Uighur	Hui
PIC	0.6744	0.6734	0.7658	0.6778	0.5491	0.5720	0.5251	0.4979
PD_f_	0.8764	0.8756	0.9295	0.8781	0.7915	0.8104	0.7704	0.7467
PD_m_	0.7191	0.7186	0.7938	0.7228	0.5874	0.6079	0.5680	0.5347
Ho	0.7410	0.6890	0.7270	0.6320	0.6780	0.6560	0.5000	0.6030
He	0.7844	0.7839	0.8660	0.7885	0.6608	0.6839	0.6390	0.6015
MEC_t_	0.6744	0.6734	0.7658	0.6778	0.5491	0.5720	0.5251	0.4979
MEC_d_	0.5343	0.5314	0.6402	0.5373	0.4006	0.4228	0.3769	0.3508

**Table 7:** Forensic parameters of 19 X-STR loci among the four ethnic populations.

	DXS8378				HPRTB			
	Han	Tibet	Uighur	Hui	Han	Tibet	Uighur	Hui
PIC	0.5510	0.6017	0.6123	0.5486	0.6734	0.6335	0.7246	0.6591
PD_f_	0.7869	0.8253	0.8315	0.7842	0.8769	0.8483	0.9059	0.8689
PD_m_	0.6191	0.6624	0.6754	0.6200	0.7157	0.6877	0.7620	0.7004
Ho	0.6600	0.6720	0.7270	0.5740	0.7410	0.6890	0.6970	0.7790
He	0.6879	0.7360	0.7505	0.6889	0.8179	0.7859	0.8710	0.8005
MEC_t_	0.5510	0.6017	0.6123	0.5486	0.6734	0.6335	0.7246	0.6591
MEC_d_	0.4048	0.4567	0.4662	0.4032	0.5312	0.4879	0.5894	0.5154

**Table 8:** Forensic parameters of 19 X-STR loci among the four ethnic populations.

	DXS101				DXS10135			
	Han	Tibet	Uighur	Hui	Han	Tibet	Uighur	Hui
PIC	0.7627	0.7795	0.8392	0.7939	0.9168	0.8875	0.9257	0.9104
PDf	0.9278	0.9357	0.9634	0.9433	0.9886	0.9804	0.9907	0.9870
PD_m_	0.7914	0.8062	0.8547	0.8172	0.9222	0.8964	0.9301	0.9165
Ho	0.7440	0.8030	0.6670	0.8240	0.8680	0.7870	0.8940	0.8820
He	0.8379	0.8536	0.9050	0.8652	0.9519	0.9254	0.9601	0.9460
MEC_t_	0.7627	0.7795	0.8392	0.7939	0.9168	0.8875	0.9257	0.9104
MEC_d_	0.6363	0.6568	0.7368	0.6755	0.8515	0.8061	0.8658	0.8414

**Table 9:** Forensic parameters of 19 X-STR loci among the four ethnic populations.

	DXS10148				DXS10101			
	Han	Tibet	Uighur	Hui	Han	Tibet	Uighur	Hui
PIC	0.8976	0.8854	0.8970	0.8850	0.8754	0.8780	0.9046	0.8717
PD_f_	0.9833	0.9796	0.9832	0.9795	0.9767	0.9775	0.9853	0.9752
PD_m_	0.9054	0.8948	0.9047	0.8943	0.8856	0.8880	0.9115	0.8828
Ho	0.8870	0.8520	0.7880	0.8090	0.8010	0.7700	0.8640	0.8380
He	0.9346	0.9236	0.9338	0.9232	0.9259	0.9284	0.9529	0.9229
MEC_t_	0.8976	0.8854	0.8970	0.8850	0.8754	0.8780	0.9046	0.8717
MEC_d_	0.8211	0.8025	0.8205	0.8020	0.7883	0.7921	0.8321	0.7825

**Table 10:** Forensic parameters of 19 X-STR loci among the four ethnic populations.

	DXS10103			
	Han	Tibet	Uighur	Hui
PIC	0.6964	0.6537	0.7202	0.7274
PD_f_	0.8897	0.8619	0.9051	0.9082
PD_m_	0.7381	0.7044	0.7553	0.7629
Ho	0.7210	0.6890	0.7120	0.7060
He	0.8303	0.7924	0.8497	0.8583
MEC_t_	0.6964	0.6537	0.7202	0.7274
MEC_d_	0.5575	0.5107	0.5846	0.5933

PIC: polymorphism information content, PD_f_: power of discrimination in females, PD_m_: power of discrimination in males, Ho: observed heterozygosity, He: expected heterozygosity, MEC_t_: trio mean exclusion chance. MEC_d_: duo mean exclusion chance Han: Southern Han.

**Table 11:** P value for LDE in four ethnic groups.

Locus by locus	Southern Han (202)	Tibet (152)	Uighur (145)	Hui (132)
Cluster I				
DXS10148-DXS10135	0.6940	0.1050	0.2490	0.0500
DXS10148-DXS8378	0.5170	0.3230	0.5750	0.9130
DXS10135-DXS8378	0.4900	0.0240	0.9420	0.2510
Cluster II				
DXS10159-DXS10162	0.0600	**0.0000**	0.4760	0.8420
DXS10159-DXS10164	0.0140	0.1240	0.3070	0.5180
DXS10162-DXS10164	0.1810	0.3060	0.0500	0.0030
Cluster III				
DXS7132-DXS10079	0.0150	0.0040	0.6710	0.2630
DXS7132-DXS10074	0.7780	0.0070	0.7080	0.0640
DXS10079-DXS10074	0.2250	**0.0000**	0.0090	0.1900
DXS10079-DXS10075	0.2470	0.5540	0.3720	0.0150
DXS10074-DXS10075	0.4850	0.0010	**0.0000**	0.0050
Cluster IV				
DXS6809-DXS6789	0.2040	**0.0000**	0.0170	0.2630
Cluster V				
DXS7424-DXS101	0.2390	0.0120	0.3960	0.2130
Cluster VI				
DXS10103-HPRTB	0.3180	0.4450	0.3700	0.0230
DXS10103-DXS10101*	**0.0000**	**0.0000**	**0.0000**	**0.0000**
HPRTB-DXS10101	0.0640	**0.0000**	0.0130	0.0840
Cluster VII				
DXS10134-DXS7423	0.1410	0.0090	0.4330	0.6210

*Indicate LDE in all four ethnic groups in China.

**Table 12:** Forensic statistical parameters of the five haplogroups.

Haplotype	Ethnic groups	PIC	He	Haplotype Diversity	PD female	PD male	MEC_t_	MEC_d_
DXS10159-DXS10162	Tibet	0.92750	0.96744	0.95931	0.99121	0.93161	0.92750	0.86913
DXS10074-DXS10075	Uighur	0.94673	0.96413	0.97787	0.99508	0.94906	0.94673	0.90159
DXS6809-DXS6789	Tibet	0.98800	0.98187	0.94327	0.99972	0.98814	0.98800	0.97647
DXS10103-DXS10101	SouthernHan	0.99080	0.96949	0.95660	0.99984	0.99088	0.99080	0.98188
	Tibet	0.98783	0.96049	0.96357	0.99971	0.98797	0.98783	0.97613
	Uighur	0.98957	0.98645	0.97261	0.99979	0.98968	0.98957	0.97950
	Hui	0.98839	0.97959	0.93199	0.99974	0.98852	0.98839	0.97720
DXS10103-HPRTB-DXS10101	Tibet	0.96412	0.98572	0.93778	0.99770	0.96520	0.96412	0.93229

PIC: Polymorphism information content, according to Desmarais, He: Expected Heterozygosity, PD_f_: power of discrimination in females, PD_m_: power of discrimination in males, MEC_t_: trio mean exclusion chance, MEC_d_: duo mean exclusion chance.

**Table 13:** Computing conventional F-Statistics from haplotype frequencies in four ethnic groups.

	Southern Han	Tibet	Uighur	Hui
Southern Han	0.00000			
P	*			
Tibet	0.00629	0.00000		
P	0.00000 ± 0.0000	*		
Uighur	0.01069	0.01142	0.00000	
P	0.00000 ± 0.0000	0.00000 ± 0.0000	*	
Hui	0.00128	0.00719	0.00896	0.00000
P	0.46847 ± 0.0572	0.00000 ± 0.0000	0.00000 ± 0.0000	*

Significance Level = 0.0500, permutations = 110, *means null.

**Table 14:** Combined Forensic efficiency parameters calculated according to both allele frequencies and haplotype frequencies of the 19 X-STR loci in four ethnic group respectively.

	X-STR + relevant linkage haplotype			
	Han	Tibet	Uighur	Hui
CPD_f_	1.000 000 000 000 000	1.000 000 000 000 000	1.000 000 000 000 000	1.000 000 000 000 000
CPD_m_	0.999 999 999 999 556	0.999 999 999 997 940	0.999 999 999 999 726	0.999 999 999 999 545
CMEC_t_	0.999 999 999 995 831	0.999 999 999 989 069	0.999 999 999 997 926	0.999 999 999 995 724
CMEC_d_	0.999 999 992 887 471	0.999 999 991 939 326	0.999 999 996 578 868	0.999 999 992 712 299

CDP_f_: combined power of discrimination in females, CDP_m_: combined power of discrimination in males, CMEC_t_: combined mean exclusion chance in trio cases, CMEC_d_: combined mean exclusion chance in duo cases, Han: Southern Han.

**Table 15:** The recombination study of 40 two-generation families with two or more children.

Marker1	Marker2	Maximum LOD score	Recombination fraction(θ)	Genetic distance (cM)	Physical distance(Mb)	95% Cls (1-LOD)
DXS10148	DXS10135	17.128	0.029	0.190	0.001	0.0035–0.0994
DXS10135	DXS8378	13.396	0.035	0.180	0.131	0.0043–0.1211
DXS8378	DXS10159	1.328	0.333	**69.800**	**47.436**	0.2109–0.4747
DXS10159	DXS10162	16.551	0.029	0.640	5.034	0.0036–0.1022
DXS10162	DXS10164	11.755	0.022	0.010	0.361	0.0005–0.1153
DXS10164	DXS7132	8.564	0.029	**0.090**	**2.411**	0.0007–0.1492
DXS7132	DXS10079	13.827	0.000	0.070	2.060	0.0000–0.0771
DXS10079	DXS10074	16.833	0.000	0.010	0.262	0.0000–0.0637
DXS10074	DXS10075	15.631	0.000	0.000	0.021	0.0000–0.0685
DXS10075	DXS6809	3.359	0.246	**17.290**	**27.910**	0.1413–0.3776
DXS6809	DXS6789	14.138	0.058	0.350	0.511	0.0160–0.1418
DXS6789	DXS7424	12.768	0.063	**6.780**	**5.169**	0.0173–0.1524
DXS7424	DXS101	15.932	0.000	0.900	0.795	0.0000–0.0672
DXS101	DXS10103	4.180	0.191	**33.220**	**31.946**	0.0915–0.3326
DXS10103	HPRTB	8.036	0.053	0.290	0.197	0.0064–0.1775
HPRTB	DXS10101	10.869	0.070	0.090	0.039	0.0194–0.1700
DXS10101	DXS10134	3.571	0.261	**34.210**	**15.919**	0.1625–0.3806
DXS10134	DXS7423	7.330	0.118	0.230	0.059	0.0444–0.2387

*Maximum LOD scores > 3 means significant linkage, The numbers of informative meioses ranged from 48 to 87, 95% Cls calculated from http://statpages.info/confint.html, The bold number mean the cM and Mb between the broder clusters.

**Table 16:** Reynolds genetic distance between populations.

\	Han	Tibet	Uighur	Hui
Han	0.00000			
Tibet	0.00631	0.00000		
Uighur	0.01075	**0.01149**	0.00000	
Hui	**0.00128**	0.00722	0.00900	0.00000

The max and min value are indicated in bold.
