# Supplementary material for: Genetic analysis of 19 X chromosome STR loci for forensic purposes in four Chinese ethnic groups
Source: Sci Rep. 2017 Feb 17;7:42782. doi: 10.1038/srep42782 (PMC5314363; doi:10.1038/srep42782)
Supplement: Supplementary TableS 1-22 [file srep42782-s1.docx]

Supplementary Table S1-S21 and Supplementary Figure S1

Genetic analysis of 19 X chromosomal STR loci for forensic purposes in four ethnics from China

Xingyi Yang^1,2^, Xiaofang Zhang^1,2a^ , Junyong Zhu^2a^, Linli Chen^3^, Changhui Liu^2^, Xingling Feng^1,2^, Ling Chen^1^, Hui Jun Wang^1^, Chao Liu^1,2*^

1. Department of Forensic Medicine, School of Basic Medical Sciences, Southern Medical University, Guangzhou, Guangdong Province 510515, PR China.

2. Guangzhou Forensic Science Institute, Guangdong Province Key Laboratory of Forensic Genetics, Guangzhou 510030,PR China.

3.AGCU ScienTch Incorporation, Wuxi 214174, PR China. Electronic

a. These authors contributed equally to this work.

* Correspondence and requests for materials should be addressed to Chao Liu (chaoliugaj123@126.com)

| **Supplementary Table S1. Allele frequencies of 19 X-STR loci among the four ethnic populations** | | | | | | | | |  |  |
| --- | --- | --- | --- | --- | --- | --- | --- | --- | --- | --- |
| **Allele** | **DXS10159** | | | |  |  | **DXS6809** | | | |
|  | **Han** | **Tibet** | **Uighur** | **Hui** |  |  | **Han** | **Tibet** | **Uighur** | **Hui** |
| 20 |  |  | 0.0047 |  |  | 25 |  | 0.0047 |  |  |
| 21 | 0.0016 |  |  |  |  | 27 |  |  |  | 0.0025 |
| 21.2 |  | 0.0047 |  |  |  | 28 |  |  | 0.0047 |  |
| 22 | 0.0049 | 0.0188 | 0.0284 |  |  | 29 | 0.0065 | 0.0047 | 0.0308 | 0.0075 |
| 23 | 0.0471 | 0.0423 | 0.0616 | 0.0500 |  | 30 | 0.0179 | 0.0047 | 0.0284 | 0.0250 |
| 24 | 0.2549 | 0.2723 | 0.3507 | 0.2975 |  | 31 | 0.1136 | 0.1221 | 0.1185 | 0.1625 |
| 24.2 |  |  |  | 0.0050 |  | 32 | 0.1347 | 0.1901 | 0.2133 | 0.1325 |
| 25 | 0.2776 | 0.2441 | 0.2085 | 0.2375 |  | 33 | 0.2873 | 0.2887 | 0.2962 | 0.2750 |
| 26 | 0.2549 | 0.2113 | 0.2062 | 0.2500 |  | 34 | 0.2662 | 0.2606 | 0.2322 | 0.2575 |
| 27 | 0.1071 | 0.1573 | 0.0924 | 0.1150 |  | 34.1 | 0.0032 |  |  |  |
| 28 | 0.0422 | 0.0376 | 0.0379 | 0.0375 |  | 35 | 0.1023 | 0.1056 | 0.0332 | 0.1050 |
| 29 | 0.0097 | 0.0117 | 0.0095 | 0.0075 |  | 36 | 0.0568 | 0.0188 | 0.0190 | 0.0275 |
|  |  |  |  |  |  | 37 | 0.0049 |  | 0.0237 | 0.0050 |
|  |  |  |  |  |  | 38 | 0.0032 |  |  |  |
|  |  |  |  |  |  | 39 | 0.0032 |  |  |  |
|  |  |  |  |  |  |  |  |  |  |  |
| 9947A | 24,25 | | | |  |  | 31,34 | | | |
|  |  |  |  |  |  |  |  |  |  |  |
| **Supplementary Table S2** | | |  |  |  |  |  |  |  |  |
| **Allele** | **DXS10134** | | | |  |  | **DXS10074** | | | |
|  | **Han** | **Tibet** | **Uighur** | **Hui** |  |  | **Han** | **Tibet** | **Uighur** | **Hui** |
| 30 | 0.0016 |  |  | 0.0050 |  | 7 |  | 0.0094 | 0.0403 | 0.0150 |
| 31 | 0.0146 | 0.0047 |  | 0.0100 |  | 8 |  |  | 0.0498 |  |
| 31.1 | 0.0016 |  |  |  |  | 12 | 0.0032 |  |  |  |
| 32 | 0.0341 | 0.0141 | 0.0284 | 0.0225 |  | 13 |  | 0.0188 | 0.0047 |  |
| 33 | 0.0487 | 0.0446 | 0.0427 | 0.0250 |  | 14 | 0.0114 | 0.0094 | 0.0071 | 0.0075 |
| 33.2 |  | 0.0047 |  |  |  | 14.3 |  | 0.0047 |  |  |
| 34 | 0.0925 | 0.0446 | 0.1019 | 0.0700 |  | 15 | 0.0373 | 0.0563 | 0.0687 | 0.0500 |
| 34.1 | 0.0032 |  |  |  |  | 15.3 |  | 0.0282 |  | 0.0050 |
| 34.2 | 0.0032 | 0.0047 | 0.0047 |  |  | 16 | 0.2062 | 0.1808 | 0.2441 | 0.2250 |
| 35 | 0.2062 | 0.1925 | 0.1872 | 0.2000 |  | 16.3 |  | 0.0141 |  |  |
| 35.1 | 0.0032 |  |  |  |  | 17 | 0.3312 | 0.2770 | 0.3128 | 0.2950 |
| 35.2 | 0.0032 | 0.0047 |  | 0.0100 |  | 18 | 0.2727 | 0.2629 | 0.1730 | 0.2525 |
| 35.3 | 0.0032 | 0.0047 |  |  |  | 19 | 0.1104 | 0.1268 | 0.0900 | 0.1300 |
| 36 | 0.1964 | 0.1596 | 0.1825 | 0.1850 |  | 20 | 0.0227 | 0.0094 | 0.0095 | 0.0150 |
| 36.1 |  |  |  | 0.0050 |  | 21 | 0.0016 | 0.0023 |  | 0.0050 |
| 36.2 |  |  | 0.0047 |  |  | 22 | 0.0032 |  |  |  |
| 36.3 | 0.0032 | 0.0094 |  |  |  |  |  |  |  |  |
| 37 | 0.1672 | 0.2723 | 0.1754 | 0.2025 |  |  |  |  |  |  |
| 37.3 | 0.0357 | 0.0305 | 0.0403 | 0.0475 |  |  |  |  |  |  |
| 38 | 0.1039 | 0.1291 | 0.0948 | 0.1025 |  |  |  |  |  |  |
| 38.3 |  | 0.0235 | 0.0332 | 0.0050 |  |  |  |  |  |  |
| 39 | 0.0568 | 0.0493 | 0.0403 | 0.0825 |  |  |  |  |  |  |
| 39.2 |  | 0.0047 |  |  |  |  |  |  |  |  |
| 39.3 | 0.0065 | 0.0023 | 0.0095 | 0.0050 |  |  |  |  |  |  |
| 40 | 0.0081 |  | 0.0024 | 0.0150 |  |  |  |  |  |  |
| 40.3 |  |  | 0.0047 | 0.0050 |  |  |  |  |  |  |
| 41 |  |  | 0.0024 |  |  |  |  |  |  |  |
| 41.3 | 0.0065 |  | 0.0142 | 0.0025 |  |  |  |  |  |  |
| 42.3 |  |  | 0.0118 |  |  |  |  |  |  |  |
| 43.3 |  |  | 0.0142 |  |  |  |  |  |  |  |
| 44.3 |  |  | 0.0047 |  |  |  |  |  |  |  |
|  |  |  |  |  |  |  |  |  |  |  |
| 9947A | 35,36 | | | |  |  | 16,19 | | | |
|  |  |  |  |  |  |  |  |  |  |  |
| **Supplementary Table S3** | | |  |  |  |  |  |  |  |  |
| **Allele** | **DXS10079** | | | |  |  | **DXS10162** | | | |
|  | **Han** | **Tibet** | **Uighur** | **Hui** |  |  | **Han** | **Tibet** | **Uighur** | **Hui** |
| 14 | 0.0016 |  | 0.0024 |  |  | 13 | 0.0032 |  | 0.0024 |  |
| 15 | 0.0016 |  | 0.0142 | 0.0100 |  | 15 | 0.0130 |  | 0.0166 | 0.0200 |
| 16 | 0.0146 | 0.0164 | 0.0308 | 0.0200 |  | 16 | 0.0455 | 0.0423 | 0.0427 | 0.0700 |
| 17 | 0.0844 | 0.0282 | 0.0782 | 0.0875 |  | 17 | 0.1656 | 0.2160 | 0.2180 | 0.1350 |
| 17.3 |  |  |  | 0.0050 |  | 18 | 0.3409 | 0.3873 | 0.3436 | 0.3375 |
| 18 | 0.1445 | 0.1056 | 0.1185 | 0.0750 |  | 19 | 0.2695 | 0.2840 | 0.2180 | 0.2700 |
| 19 | 0.2094 | 0.2559 | 0.2962 | 0.2700 |  | 20 | 0.1250 | 0.0610 | 0.1161 | 0.1450 |
| 20 | 0.2857 | 0.2934 | 0.2512 | 0.2300 |  | 21 | 0.0341 | 0.0094 | 0.0355 | 0.0175 |
| 21 | 0.1591 | 0.2089 | 0.1327 | 0.2000 |  | 22 | 0.0032 |  | 0.0047 | 0.0050 |
| 22 | 0.0714 | 0.0681 | 0.0735 | 0.0725 |  | 23 |  |  | 0.0024 |  |
| 23 | 0.0260 | 0.0188 | 0.0024 | 0.0250 |  |  |  |  |  |  |
| 24 | 0.0016 | 0.0047 |  | 0.0050 |  |  |  |  |  |  |
|  |  |  |  |  |  |  |  |  |  |  |
| 9947A | 20,23 | | | |  |  | 19,19 | | | |
|  |  |  |  |  |  |  |  |  |  |  |
| **Supplementary Table S4** | | |  |  |  |  |  |  |  |  |
| **Allele** | **DXS6789** | | | |  |  | **DXS10075** | | | |
|  | **Han** | **Tibet** | **Uighur** | **Hui** |  |  | **Han** | **Tibet** | **Uighur** | **Hui** |
| 14 | 0.0049 | 0.0094 | 0.0024 |  |  | 13 |  | 0.0094 | 0.0355 | 0.0150 |
| 15 | 0.1039 | 0.1714 | 0.0427 | 0.1600 |  | 14 |  | 0.0047 |  | 0.0025 |
| 16 | 0.3409 | 0.1737 | 0.1422 | 0.3050 |  | 15 | 0.0130 | 0.0047 | 0.0071 | 0.0100 |
| 17 | 0.0601 | 0.0023 | 0.0118 | 0.0500 |  | 16 | 0.2354 | 0.2042 | 0.1635 | 0.2525 |
| 18 | 0.0081 |  | 0.0047 | 0.0050 |  | 16.2 | 0.0260 | 0.0094 | 0.0190 | 0.0075 |
| 19 | 0.0211 | 0.0915 | 0.0640 | 0.0275 |  | 17 | 0.4010 | 0.4531 | 0.3886 | 0.3800 |
| 20 | 0.2240 | 0.2793 | 0.2607 | 0.1975 |  | 17.2 | 0.0260 | 0.0258 | 0.0142 | 0.0225 |
| 21 | 0.1753 | 0.1995 | 0.2488 | 0.1825 |  | 18 | 0.2565 | 0.2512 | 0.3175 | 0.2850 |
| 22 | 0.0519 | 0.0563 | 0.1825 | 0.0675 |  | 18.2 | 0.0016 | 0.0047 | 0.0071 | 0.0100 |
| 23 | 0.0065 | 0.0070 | 0.0332 | 0.0050 |  | 19 | 0.0325 | 0.0282 | 0.0474 | 0.0150 |
| 24 | 0.0032 | 0.0094 | 0.0071 |  |  | 19.2 | 0.0049 |  |  |  |
|  |  |  |  |  |  | 20 | 0.0032 | 0.0047 |  |  |
|  |  |  |  |  |  |  |  |  |  |  |
| 9947A | 21,22 | | | |  |  | 17,18 | | | |
|  |  |  |  |  |  |  |  |  |  |  |
| **Supplementary Table S5** | | |  |  |  |  |  |  |  |  |
| **Allele** | **DXS7132** | | | |  |  | **DXS7423** | | | |
|  | **Han** | **Tibet** | **Uighur** | **Hui** |  |  | **Han** | **Tibet** | **Uighur** | **Hui** |
| 11 | 0.0065 | 0.0141 | 0.0047 |  |  | 11 |  | 0.0047 |  |  |
| 12 | 0.0714 | 0.0845 | 0.0995 | 0.0900 |  | 13 | 0.0016 |  | 0.0545 | 0.0050 |
| 13 | 0.1737 | 0.1784 | 0.3104 | 0.2100 |  | 14 | 0.3539 | 0.4085 | 0.2701 | 0.3275 |
| 14 | 0.3734 | 0.4484 | 0.3318 | 0.3575 |  | 15 | 0.5942 | 0.5446 | 0.4763 | 0.6125 |
| 15 | 0.2744 | 0.2113 | 0.2014 | 0.2800 |  | 16 | 0.0438 | 0.0376 | 0.1730 | 0.0475 |
| 16 | 0.0844 | 0.0469 | 0.0427 | 0.0550 |  | 17 | 0.0065 | 0.0047 | 0.0213 | 0.0050 |
| 17 | 0.0130 | 0.0117 | 0.0095 | 0.0075 |  | 18 |  |  | 0.0047 |  |
| 18 | 0.0032 | 0.0047 |  |  |  | 20 |  |  |  | 0.0025 |
|  |  |  |  |  |  |  |  |  |  |  |
| 9947A | 12,12 | | | |  |  | 14,15 | | | |
|  |  |  |  |  |  |  |  |  |  |  |
|  |  |  |  |  |  |  |  |  |  |  |
| **Supplementary Table S6** | | |  |  |  |  |  |  |  |  |
| **Allele** | **DXS7424** | | | |  |  | **DXS10164** | | | |
|  | **Han** | **Tibet** | **Uighur** | **Hui** |  |  | **Han** | **Tibet** | **Uighur** | **Hui** |
| 8 |  | 0.0023 |  |  |  | 5 |  | 0.0047 |  |  |
| 9 | 0.0065 |  |  |  |  | 8 | 0.0276 | 0.0751 | 0.0166 | 0.0525 |
| 10 | 0.0049 |  | 0.0047 |  |  | 9 | 0.0390 | 0.0305 | 0.0355 | 0.0150 |
| 11 | 0.0130 | 0.0047 | 0.0332 | 0.0150 |  | 10 | 0.6006 | 0.5845 | 0.6137 | 0.6500 |
| 12 | 0.0097 | 0.0047 | 0.0213 | 0.0100 |  | 11 | 0.2029 | 0.1761 | 0.2014 | 0.1850 |
| 13 | 0.0308 | 0.0305 | 0.0545 | 0.0250 |  | 12 | 0.0844 | 0.1127 | 0.1137 | 0.0725 |
| 14 | 0.1071 | 0.0704 | 0.1682 | 0.1525 |  | 13 | 0.0325 | 0.0117 | 0.0166 | 0.0175 |
| 15 | 0.3571 | 0.2582 | 0.2464 | 0.3750 |  | 14 | 0.0130 |  | 0.0024 | 0.0075 |
| 16 | 0.3653 | 0.4085 | 0.3152 | 0.3275 |  | 15 |  | 0.0047 |  |  |
| 17 | 0.0828 | 0.2042 | 0.1066 | 0.0650 |  |  |  |  |  |  |
| 18 | 0.0179 | 0.0164 | 0.0427 | 0.0300 |  |  |  |  |  |  |
| 19 | 0.0049 |  | 0.0071 |  |  |  |  |  |  |  |
|  |  |  |  |  |  |  |  |  |  |  |
| 9947A | 14,16 | | | |  |  | 10,10 | | | |
|  |  |  |  |  |  |  |  |  |  |  |
| **Supplementary Table S7** | | |  |  |  |  |  |  |  |  |
| **Allele** | **DXS8378** | | | |  |  | **HPRTB** | | | |
|  | **Han** | **Tibet** | **Uighur** | **Hui** |  |  | **Han** | **Tibet** | **Uighur** | **Hui** |
| 7.2 |  | 0.0141 |  |  |  | 9 | 0.0032 | 0.0023 | 0.0142 |  |
| 8 |  |  | 0.0047 |  |  | 11 | 0.0828 | 0.0188 | 0.0995 | 0.0900 |
| 8.2 |  | 0.0094 |  |  |  | 12 | 0.2403 | 0.2136 | 0.2512 | 0.2350 |
| 9 | 0.0195 | 0.0117 | 0.0284 | 0.0225 |  | 12.3 | 0.0032 |  |  |  |
| 9.2 |  | 0.0094 |  |  |  | 13 | 0.4302 | 0.4343 | 0.3389 | 0.4600 |
| 10 | 0.5049 | 0.4343 | 0.3768 | 0.4875 |  | 14 | 0.1786 | 0.2746 | 0.2109 | 0.1500 |
| 11 | 0.3295 | 0.3638 | 0.3791 | 0.3575 |  | 15 | 0.0519 | 0.0469 | 0.0735 | 0.0425 |
| 12 | 0.1299 | 0.1221 | 0.1943 | 0.1175 |  | 16 | 0.0097 | 0.0094 |  | 0.0175 |
| 13 | 0.0130 | 0.0352 | 0.0166 | 0.0150 |  |  |  |  |  |  |
| 14 | 0.0032 |  |  |  |  |  |  |  |  |  |
|  |  |  |  |  |  |  |  |  |  |  |
| 9947A | 10,11 | | | |  |  | 14,14 | | | |
|  |  |  |  |  |  |  |  |  |  |  |
| **Supplementary Table S8** | | |  |  |  |  |  |  |  |  |
| **Allele** | **DXS101** | | | |  |  | **DXS10135** | | | |
|  | **Han** | **Tibet** | **Uighur** | **Hui** |  |  | **Han** | **Tibet** | **Uighur** | **Hui** |
| 15 |  |  | 0.0166 |  |  | 16 |  |  | 0.0024 | 0.0025 |
| 16 |  |  | 0.0047 |  |  | 17 | 0.0097 | 0.0070 | 0.0024 | 0.0075 |
| 18 |  |  | 0.0427 | 0.0050 |  | 18 | 0.0195 | 0.0657 | 0.0284 | 0.0125 |
| 19 |  |  | 0.0142 | 0.0025 |  | 19 | 0.0731 | 0.1268 | 0.0782 | 0.0800 |
| 20 | 0.0032 |  | 0.0190 |  |  | 19.1 |  |  | 0.0213 |  |
| 20.2 |  |  |  | 0.0050 |  | 20 | 0.1136 | 0.1455 | 0.1137 | 0.0650 |
| 21 | 0.0065 | 0.0047 | 0.0166 | 0.0100 |  | 20.1 |  |  | 0.0095 |  |
| 22 | 0.0292 | 0.0352 | 0.0213 | 0.0350 |  | 21 | 0.1136 | 0.1362 | 0.0735 | 0.1500 |
| 23 | 0.1006 | 0.1033 | 0.1209 | 0.1225 |  | 22 | 0.1104 | 0.1596 | 0.0948 | 0.1025 |
| 24 | 0.3133 | 0.2512 | 0.2133 | 0.2925 |  | 22.1 | 0.0032 |  | 0.0024 |  |
| 25 | 0.2581 | 0.1901 | 0.2346 | 0.1825 |  | 23 | 0.0958 | 0.0892 | 0.0853 | 0.0900 |
| 26 | 0.1575 | 0.2700 | 0.1327 | 0.1950 |  | 23.1 |  | 0.0047 |  |  |
| 27 | 0.0828 | 0.0939 | 0.0735 | 0.0900 |  | 24 | 0.0812 | 0.0728 | 0.0616 | 0.0975 |
| 28 | 0.0325 | 0.0211 | 0.0569 | 0.0350 |  | 24.1 |  | 0.0047 |  |  |
| 29 | 0.0065 | 0.0141 | 0.0213 | 0.0100 |  | 25 | 0.0422 | 0.0352 | 0.0735 | 0.0950 |
| 30 | 0.0065 | 0.0164 | 0.0071 | 0.0100 |  | 26 | 0.0487 | 0.0352 | 0.0616 | 0.0400 |
| 31 |  |  | 0.0047 | 0.0050 |  | 27 | 0.0795 | 0.0023 | 0.0640 | 0.0350 |
| 32 | 0.0032 |  |  |  |  | 28 | 0.0633 | 0.0188 | 0.0829 | 0.0650 |
|  |  |  |  |  |  | 29 | 0.0227 | 0.0305 | 0.0450 | 0.0550 |
|  |  |  |  |  |  | 30 | 0.0390 | 0.0164 | 0.0355 | 0.0450 |
|  |  |  |  |  |  | 30.2 |  |  | 0.0024 |  |
|  |  |  |  |  |  | 31 | 0.0357 | 0.0164 | 0.0118 | 0.0175 |
|  |  |  |  |  |  | 31.2 |  | 0.0047 |  |  |
|  |  |  |  |  |  | 32 | 0.0162 | 0.0117 | 0.0118 | 0.0225 |
|  |  |  |  |  |  | 33 | 0.0049 | 0.0117 | 0.0071 | 0.0100 |
|  |  |  |  |  |  | 34 | 0.0081 |  | 0.0095 |  |
|  |  |  |  |  |  | 35 | 0.0065 |  | 0.0095 |  |
|  |  |  |  |  |  | 35.2 | 0.0016 |  |  |  |
|  |  |  |  |  |  | 36 | 0.0032 |  | 0.0024 | 0.0050 |
|  |  |  |  |  |  | 36.2 | 0.0032 |  |  |  |
|  |  |  |  |  |  | 37 | 0.0016 |  |  | 0.0025 |
|  |  |  |  |  |  | 38 | 0.0032 | 0.0047 | 0.0095 |  |
|  |  |  |  |  |  |  |  |  |  |  |
| 9947A | 24,26 | | | |  |  | 21.1,27 | | | |
|  |  |  |  |  |  |  |  |  |  |  |
| **Supplementary Table S9** | | |  |  |  |  |  |  |  |  |
| **Allele** | **DXS10148** | | | |  |  | **DXS10101** | | | |
|  | **Han** | **Tibet** | **Uighur** | **Hui** |  |  | **Han** | **Tibet** | **Uighur** | **Hui** |
| 17 |  |  | 0.0047 | 0.0025 |  | 26 | 0.0016 |  |  |  |
| 18 | 0.1282 | 0.1620 | 0.1232 | 0.1450 |  | 26.2 |  | 0.0094 | 0.0071 | 0.0050 |
| 19 | 0.0227 | 0.0235 | 0.0687 | 0.0325 |  | 27 | 0.0162 |  | 0.0095 |  |
| 19.1 | 0.0032 |  |  | 0.0025 |  | 27.2 | 0.0049 |  | 0.0308 |  |
| 20 | 0.0114 | 0.0070 | 0.0213 | 0.0125 |  | 28 | 0.0114 | 0.0211 | 0.0237 | 0.0050 |
| 20.1 | 0.0081 |  | 0.0142 |  |  | 28.2 | 0.0179 | 0.0516 | 0.0995 | 0.0225 |
| 21 | 0.0065 | 0.0047 |  | 0.0050 |  | 29 | 0.0227 | 0.0282 | 0.0284 | 0.0225 |
| 21.1 | 0.0211 | 0.0164 | 0.0024 | 0.0025 |  | 29.2 | 0.0373 | 0.0329 | 0.0972 | 0.0850 |
| 22 |  |  | 0.0024 |  |  | 30 | 0.0974 | 0.0446 | 0.0569 | 0.1150 |
| 22.1 | 0.0617 | 0.0657 | 0.0332 | 0.0300 |  | 30.2 | 0.0747 | 0.0634 | 0.1185 | 0.0625 |
| 23 |  |  | 0.0213 |  |  | 30.3 | 0.0016 |  |  |  |
| 23.1 | 0.1006 | 0.0751 | 0.0498 | 0.0975 |  | 31 | 0.1867 | 0.1878 | 0.1398 | 0.1875 |
| 24 |  |  | 0.0095 |  |  | 31.2 | 0.1055 | 0.1714 | 0.1043 | 0.1325 |
| 24.1 | 0.1055 | 0.0587 | 0.1090 | 0.1450 |  | 31.3 |  |  |  | 0.0050 |
| 24.2 |  |  | 0.0024 |  |  | 32 | 0.1899 | 0.1221 | 0.0924 | 0.1700 |
| 25 |  |  | 0.0047 |  |  | 32.2 | 0.0698 | 0.0845 | 0.0711 | 0.0275 |
| 25.1 | 0.1201 | 0.1221 | 0.1114 | 0.0925 |  | 33 | 0.0893 | 0.1103 | 0.0616 | 0.0825 |
| 26.1 | 0.1218 | 0.1315 | 0.1374 | 0.1575 |  | 33.2 | 0.0308 | 0.0376 | 0.0190 | 0.0175 |
| 26.2 |  | 0.0070 |  |  |  | 34 | 0.0260 | 0.0211 | 0.0355 | 0.0475 |
| 27.1 | 0.1169 | 0.1103 | 0.1493 | 0.1050 |  | 34.2 | 0.0016 |  |  | 0.0050 |
| 27.2 | 0.0016 |  | 0.0047 | 0.0150 |  | 35 | 0.0114 | 0.0141 | 0.0047 | 0.0050 |
| 28.1 | 0.0747 | 0.1315 | 0.0450 | 0.0600 |  | 36 | 0.0032 |  |  |  |
| 28.2 | 0.0032 |  | 0.0024 | 0.0075 |  | 36.2 |  |  |  | 0.0025 |
| 28.3 | 0.0032 |  |  |  |  |  |  |  |  |  |
| 29.1 | 0.0487 | 0.0493 | 0.0521 | 0.0250 |  |  |  |  |  |  |
| 29.2 |  |  | 0.0024 |  |  |  |  |  |  |  |
| 30.1 | 0.0308 | 0.0211 | 0.0166 | 0.0550 |  |  |  |  |  |  |
| 30.2 |  |  | 0.0047 |  |  |  |  |  |  |  |
| 31.1 | 0.0032 | 0.0117 | 0.0071 | 0.0050 |  |  |  |  |  |  |
| 32.1 | 0.0016 | 0.0023 |  |  |  |  |  |  |  |  |
| 32.2 | 0.0032 |  |  |  |  |  |  |  |  |  |
| 33.1 | 0.0016 |  |  | 0.0025 |  |  |  |  |  |  |
|  |  |  |  |  |  |  |  |  |  |  |
| 9947A | 22.1,23.1 | | | |  |  | 30,31 | | | |
|  |  |  |  |  |  |  |  |  |  |  |
| **Supplementary Table S10** | | |  |  |  |  |  |  |  |  |
| **Allele** | **DXS10103** | | | |  |  |  |  |  |  |
|  | **Han** | **Tibet** | **Uighur** | **Hui** |  |  |  |  |  |  |
| 14 | 0.0065 |  |  |  |  |  |  |  |  |  |
| 15 | 0.0097 | 0.0141 | 0.0427 | 0.0225 |  |  |  |  |  |  |
| 16 | 0.3653 | 0.3850 | 0.1967 | 0.2800 |  |  |  |  |  |  |
| 17 | 0.1023 | 0.0493 | 0.0900 | 0.0925 |  |  |  |  |  |  |
| 18 | 0.1494 | 0.1526 | 0.2133 | 0.1600 |  |  |  |  |  |  |
| 19 | 0.3036 | 0.3451 | 0.3815 | 0.3425 |  |  |  |  |  |  |
| 19.2 |  |  |  | 0.0050 |  |  |  |  |  |  |
| 20 | 0.0584 | 0.0493 | 0.0711 | 0.0800 |  |  |  |  |  |  |
| 21 | 0.0049 | 0.0047 | 0.0047 | 0.0175 |  |  |  |  |  |  |
|  |  |  |  |  |  |  |  |  |  |  |
| 9947A | 17,17 | | | |  |  |  |  |  |  |
|  |  |  |  |  |  |  |  |  |  |  |

Han: Southern Han

| **Supplementary Table S11. Haplotype frequencies of DXS10159-DXS10162 in Tibet males** | | | |
| --- | --- | --- | --- |
| **No.** | **Haplotype** | **Tibet 152** | |
|  |  | **No.** | **Freq.** |
| 1 | **25-18** | 18 | 0.1184 |
| 2 | **24-18** | 15 | 0.0987 |
| 3 | **26-18** | 15 | 0.0987 |
| 4 | **24-19** | 14 | 0.0921 |
| 5 | **25-19** | 12 | 0.0789 |
| 6 | **24-17** | 11 | 0.0724 |
| 7 | **27-17** | 10 | 0.0658 |
| 8 | **26-17** | 8 | 0.0526 |
| 9 | **27-19** | 7 | 0.0461 |
| 10 | **24-20** | 5 | 0.0329 |
| 11 | **28-18** | 4 | 0.0263 |
| 12 | **27-16** | 4 | 0.0263 |
| 13 | **27-18** | 4 | 0.0263 |
| 14 | **25-17** | 4 | 0.0263 |
| 15 | **26-19** | 3 | 0.0197 |
| 16 | **26-16** | 3 | 0.0197 |
| 17 | **23-19** | 3 | 0.0197 |
| 18 | **25-20** | 2 | 0.0132 |
| 19 | **22-19** | 2 | 0.0132 |
| 20 | **26-20** | 1 | 0.0066 |
| 21 | **21.2-19** | 1 | 0.0066 |
| 22 | **25-16** | 1 | 0.0066 |
| 23 | **23-18** | 1 | 0.0066 |
| 24 | **23-21** | 1 | 0.0066 |
| 25 | **23-17** | 1 | 0.0066 |
| 26 | **29-17** | 1 | 0.0066 |
| 27 | **28-16** | 1 | 0.0066 |

| **Supplementary Table S12. Haplotype frequencies of DXS10074-DXS10075 in Uighur males** | | | |
| --- | --- | --- | --- |
| **No.** | **Haplotype** | **Uighur 145** | |
|  |  | **No.** | **Freq.** |
| 1 | **17-17** | 22 | 0.1517 |
| 2 | **17-18** | 19 | 0.1310 |
| 3 | **16-18** | 13 | 0.0897 |
| 4 | **18-18** | 10 | 0.0690 |
| 5 | **16-16** | 9 | 0.0621 |
| 6 | **16-17** | 8 | 0.0552 |
| 7 | **19-17** | 8 | 0.0552 |
| 8 | **18-17** | 7 | 0.0483 |
| 9 | **7-13** | 6 | 0.0414 |
| 10 | **15-17** | 6 | 0.0414 |
| 11 | **8-17** | 6 | 0.0414 |
| 12 | **17-19** | 4 | 0.0276 |
| 13 | **18-16** | 4 | 0.0276 |
| 14 | **17-16** | 4 | 0.0276 |
| 15 | **16-19** | 3 | 0.0207 |
| 16 | **19-16.2** | 2 | 0.0138 |
| 17 | **19-16** | 2 | 0.0138 |
| 18 | **8-18** | 2 | 0.0138 |
| 19 | **15-16** | 2 | 0.0138 |
| 20 | **19-18** | 2 | 0.0138 |
| 21 | **8-16** | 2 | 0.0138 |
| 22 | **17-16.2** | 1 | 0.0069 |
| 23 | **16-15** | 1 | 0.0069 |
| 24 | **15-18** | 1 | 0.0069 |
| 25 | **18-16.2** | 1 | 0.0069 |

| **Supplementary Table S13. Haplotype frequencies of DXS6809-DXS6789 in Tibet males** | | | |
| --- | --- | --- | --- |
| **No.** | **Haplotype** | **Tibet 152** | |
|  |  | **No.** | **Freq.** |
| 1 | **33-20** | 13 | 0.0855 |
| 2 | **32-20** | 12 | 0.0789 |
| 3 | **33-21** | 11 | 0.0724 |
| 4 | **32-21** | 10 | 0.0658 |
| 5 | **33-15** | 9 | 0.0592 |
| 6 | **34-15** | 9 | 0.0592 |
| 7 | **34-16** | 8 | 0.0526 |
| 8 | **35-16** | 6 | 0.0395 |
| 9 | **34-20** | 6 | 0.0395 |
| 10 | **34-22** | 6 | 0.0395 |
| 11 | **33-16** | 5 | 0.0329 |
| 12 | **32-15** | 5 | 0.0329 |
| 13 | **35-20** | 5 | 0.0329 |
| 14 | **33-22** | 4 | 0.0263 |
| 15 | **33-19** | 4 | 0.0263 |
| 16 | **34-19** | 4 | 0.0263 |
| 17 | **34-21** | 4 | 0.0263 |
| 18 | **31-21** | 4 | 0.0263 |
| 19 | **31-16** | 4 | 0.0263 |
| 20 | **35-19** | 4 | 0.0263 |
| 21 | **31-20** | 3 | 0.0197 |
| 22 | **36-21** | 2 | 0.0132 |
| 23 | **32-16** | 2 | 0.0132 |
| 24 | **31-19** | 2 | 0.0132 |
| 25 | **25-20** | 1 | 0.0066 |
| 26 | **31-14** | 1 | 0.0066 |
| 27 | **31-15** | 1 | 0.0066 |
| 28 | **31-22** | 1 | 0.0066 |
| 29 | **35-14** | 1 | 0.0066 |
| 30 | **35-15** | 1 | 0.0066 |
| 31 | **30-24** | 1 | 0.0066 |
| 32 | **31-24** | 1 | 0.0066 |
| 33 | **32-19** | 1 | 0.0066 |
| 34 | **29-23** | 1 | 0.0066 |

| **Supplementary Table S14. Haplotype frequencies of DXS10103-DXS10101 in all four ethnic groups males** | | | | | | | | | |
| --- | --- | --- | --- | --- | --- | --- | --- | --- | --- |
| **No.** | **Haplotype** | **Han 202** | | **Tibet 152** | | **Uighur 145** | | **Hui 132** | |
|  |  | **No.** | **Freq.** | **No.** | **Freq.** | **No.** | **Freq.** | **No.** | **Freq.** |
| 1 | **16-31** | 23 | 0.1139 | 26 | 0.1711 | 9 | 0.0621 | 12 | 0.0909 |
| 2 | **16-32** | 23 | 0.1139 | 9 | 0.0592 | 2 | 0.0138 | 10 | 0.0758 |
| 3 | **19-31.2** | 12 | 0.0594 | 14 | 0.0921 | 7 | 0.0483 | 10 | 0.0758 |
| 4 | **19-32.2** | 9 | 0.0446 | 9 | 0.0592 | 7 | 0.0483 | 3 | 0.0227 |
| 5 | **16-33** | 8 | 0.0396 | 12 | 0.0789 | 3 | 0.0207 | 5 | 0.0379 |
| 6 | **19-29.2** | 5 | 0.0248 | 3 | 0.0197 | 9 | 0.0621 | 10 | 0.0758 |
| 7 | **16-30** | 13 | 0.0644 | 4 | 0.0263 | 3 | 0.0207 | 5 | 0.0379 |
| 8 | **19-30.2** | 4 | 0.0198 | 7 | 0.0461 | 9 | 0.0621 | 4 | 0.0303 |
| 9 | **18-31.2** | 5 | 0.0248 | 6 | 0.0395 | 5 | 0.0345 | 2 | 0.0152 |
| 10 | **19-31** | 8 | 0.0396 | 2 | 0.0132 | 4 | 0.0276 | 3 | 0.0227 |
| 11 | **19-28.2** |  |  | 6 | 0.0395 | 10 | 0.0690 | 1 | 0.0076 |
| 12 | **17-31** | 5 | 0.0248 | 1 | 0.0066 | 5 | 0.0345 | 5 | 0.0379 |
| 13 | **19-32** | 7 | 0.0347 | 3 | 0.0197 | 3 | 0.0207 | 3 | 0.0227 |
| 14 | **18-30.2** | 5 | 0.0248 |  |  | 5 | 0.0345 | 4 | 0.0303 |
| 15 | **17-32** | 8 | 0.0396 | 3 | 0.0197 | 1 | 0.0069 | 1 | 0.0076 |
| 16 | **18-29.2** | 3 | 0.0149 | 2 | 0.0132 | 5 | 0.0345 | 3 | 0.0227 |
| 17 | **18-31** | 1 | 0.0050 | 1 | 0.0066 | 3 | 0.0207 | 7 | 0.0530 |
| 18 | **18-32.2** | 5 | 0.0248 | 3 | 0.0197 | 3 | 0.0207 | 1 | 0.0076 |
| 19 | **20-31.2** | 2 | 0.0099 | 3 | 0.0197 | 1 | 0.0069 | 5 | 0.0379 |
| 20 | **19-30** | 3 | 0.0149 | 1 | 0.0066 | 1 | 0.0069 | 6 | 0.0455 |
| 21 | **16-34** | 3 | 0.0149 | 2 | 0.0132 | 2 | 0.0138 | 3 | 0.0227 |
| 22 | **18-33** | 1 | 0.0050 | 4 | 0.0263 | 4 | 0.0276 |  |  |
| 23 | **20-32** | 2 | 0.0099 | 1 | 0.0066 | 2 | 0.0138 | 3 | 0.0227 |
| 24 | **19-33** | 5 | 0.0248 |  |  |  |  | 3 | 0.0227 |
| 25 | **16-29** | 1 | 0.0050 | 3 | 0.0197 | 2 | 0.0138 | 1 | 0.0076 |
| 26 | **17-30** | 3 | 0.0149 | 1 | 0.0066 | 2 | 0.0138 | 1 | 0.0076 |
| 27 | **19-33.2** | 1 | 0.0050 | 4 | 0.0263 | 1 | 0.0069 | 1 | 0.0076 |
| 28 | **18-33.2** | 3 | 0.0149 | 1 | 0.0066 | 1 | 0.0069 | 1 | 0.0076 |
| 29 | **15-33** | 1 | 0.0050 | 1 | 0.0066 | 3 | 0.0207 | 1 | 0.0076 |
| 30 | **18-28.2** | 1 | 0.0050 | 1 | 0.0066 | 2 | 0.0138 | 2 | 0.0152 |
| 31 | **20-30.2** | 1 | 0.0050 | 1 | 0.0066 | 3 | 0.0207 |  |  |
| 32 | **16-31.2** |  |  | 3 | 0.0197 | 2 | 0.0138 |  |  |
| 33 | **19-28** |  |  | 2 | 0.0132 | 3 | 0.0207 |  |  |
| 34 | **20-32.2** | 2 | 0.0099 |  |  | 1 | 0.0069 | 1 | 0.0076 |
| 35 | **18-32** |  |  | 2 | 0.0132 | 2 | 0.0138 |  |  |
| 36 | **19-34** | 3 | 0.0149 | 1 | 0.0066 |  |  |  |  |
| 37 | **15-32** | 1 | 0.0050 |  |  | 1 | 0.0069 | 2 | 0.0152 |
| 38 | **16-35** | 2 | 0.0099 | 1 | 0.0066 | 1 | 0.0069 |  |  |
| 39 | **18-30** | 1 | 0.0050 |  |  | 1 | 0.0069 | 2 | 0.0152 |
| 40 | **19-26.2** |  |  | 2 | 0.0132 | 1 | 0.0069 | 1 | 0.0076 |
| 41 | **20-27.2** | 1 | 0.0050 |  |  | 2 | 0.0138 |  |  |
| 42 | **16-28** | 2 | 0.0099 | 1 | 0.0066 |  |  |  |  |
| 43 | **16-28.2** |  |  |  |  | 3 | 0.0207 |  |  |
| 44 | **19-27** | 2 | 0.0099 |  |  | 1 | 0.0069 |  |  |
| 45 | **15-34** |  |  |  |  | 2 | 0.0138 | 1 | 0.0076 |
| 46 | **20-28.2** | 1 | 0.0050 | 2 | 0.0132 |  |  |  |  |
| 47 | **18-34** |  |  |  |  | 2 | 0.0138 |  |  |
| 48 | **19-29** |  |  | 1 | 0.0066 |  |  | 1 | 0.0076 |
| 49 | **17-30.2** | 1 | 0.0050 |  |  | 1 | 0.0069 |  |  |
| 50 | **21-31.2** |  |  |  |  |  |  | 2 | 0.0152 |
| 51 | **20-33.2** | 1 | 0.0050 |  |  |  |  | 1 | 0.0076 |
| 52 | **19-27.2** |  |  |  |  | 2 | 0.0138 |  |  |
| 53 | **20-30** | 1 | 0.0050 |  |  |  |  | 1 | 0.0076 |
| 54 | **20-28** |  |  |  |  | 1 | 0.0069 | 1 | 0.0076 |
| 55 | **17-33** | 1 | 0.0050 |  |  |  |  |  |  |
| 56 | **17-32.2** | 1 | 0.0050 |  |  |  |  |  |  |
| 57 | **17-33.2** | 1 | 0.0050 |  |  |  |  |  |  |
| 58 | **21-32** |  |  | 1 | 0.0066 |  |  |  |  |
| 59 | **21-29** |  |  |  |  |  |  | 1 | 0.0076 |
| 60 | **21-32.2** | 1 | 0.0050 |  |  |  |  |  |  |
| 61 | **14-31** | 1 | 0.0050 |  |  |  |  |  |  |
| 62 | **14-32** | 1 | 0.00495 |  |  |  |  |  |  |
| 63 | **16-30.2** | 1 | 0.0050 |  |  |  |  |  |  |
| 64 | **19-35** | 1 | 0.0050 |  |  |  |  |  |  |
| 65 | **16-33.2** | 1 | 0.0050 |  |  |  |  |  |  |
| 66 | **20-29** | 1 | 0.0050 |  |  |  |  |  |  |
| 67 | **18-35** |  |  | 1 | 0.00658 |  |  |  |  |
| 68 | **16-29.2** | 1 | 0.0050 |  |  |  |  |  |  |
| 69 | **16-36** | 1 | 0.0050 |  |  |  |  |  |  |
| 70 | **18-27.2** |  |  |  |  | 1 | 0.0069 |  |  |
| 71 | **19.2-32** |  |  |  |  |  |  | 1 | 0.0076 |
| 72 | **17-35** |  |  |  |  |  |  | 1 | 0.0076 |
| 73 | **18-29** | 1 | 0.0050 |  |  |  |  |  |  |
| 74 | **15-35** |  |  | 1 | 0.0066 |  |  |  |  |
| 75 | **18-28** |  |  |  |  | 1 | 0.0069 |  |  |

Han: Southern Han

| **Supplementary Table S15. Haplotype frequencies of DXS10103-HPRTB-DXS10101 in Tibet males** | | | |
| --- | --- | --- | --- |
| **No.** | **Haplotype** | **Tibet 152** | |
|  |  | **No.** | **Freq.** |
| 1 | **16-13-31** | 14 | 0.0921 |
| 2 | **19-14-31.2** | 8 | 0.0526 |
| 3 | **16-14-31** | 8 | 0.0526 |
| 4 | **16-13-33** | 8 | 0.0526 |
| 5 | **19-13-30.2** | 5 | 0.0329 |
| 6 | **19-12-28.2** | 5 | 0.0329 |
| 7 | **18-13-33** | 4 | 0.0263 |
| 8 | **16-12-32** | 4 | 0.0263 |
| 9 | **16-14-33** | 4 | 0.0263 |
| 10 | **19-14-32.2** | 3 | 0.0197 |
| 11 | **19-13-31.2** | 3 | 0.0197 |
| 12 | **18-14-31.2** | 3 | 0.0197 |
| 13 | **19-13-33.2** | 3 | 0.0197 |
| 14 | **16-13-32** | 3 | 0.0197 |
| 15 | **19-13-32.2** | 3 | 0.0197 |
| 16 | **19-12-32** | 3 | 0.0197 |
| 17 | **18-13-32.2** | 3 | 0.0197 |
| 18 | **16-12-31** | 3 | 0.0197 |
| 19 | **16-13-31.2** | 2 | 0.0132 |
| 20 | **18-13-29.2** | 2 | 0.0132 |
| 21 | **19-12-32.2** | 2 | 0.0132 |
| 22 | **16-12-29** | 2 | 0.0132 |
| 23 | **16-12-30** | 2 | 0.0132 |
| 24 | **17-13-32** | 2 | 0.0132 |
| 25 | **19-12-31.2** | 2 | 0.0132 |
| 26 | **19-12-29.2** | 2 | 0.0132 |
| 27 | **20-14-31.2** | 2 | 0.0132 |
| 28 | **19-12-26.2** | 2 | 0.0132 |
| 29 | **16-14-30** | 2 | 0.0132 |
| 30 | **16-14-32** | 2 | 0.0132 |
| 31 | **18-15-31.2** | 2 | 0.0132 |
| 32 | **19-11-31** | 2 | 0.0132 |
| 33 | **21-13-32** | 1 | 0.0066 |
| 34 | **15-13-35** | 1 | 0.0066 |
| 35 | **16-12-31.2** | 1 | 0.0066 |
| 36 | **19-13-30** | 1 | 0.0066 |
| 37 | **19-14-30.2** | 1 | 0.0066 |
| 38 | **19-11-28** | 1 | 0.0066 |
| 39 | **19-15-29** | 1 | 0.0066 |
| 40 | **18-12-31** | 1 | 0.0066 |
| 41 | **18-13-32** | 1 | 0.0066 |
| 42 | **19-13-29.2** | 1 | 0.0066 |
| 43 | **16-14-34** | 1 | 0.0066 |
| 44 | **16-12-28** | 1 | 0.0066 |
| 45 | **19-16-34** | 1 | 0.0066 |
| 46 | **16-14-29** | 1 | 0.0066 |
| 47 | **19-15-28** | 1 | 0.0066 |
| 48 | **16-13-35** | 1 | 0.0066 |
| 49 | **15-13-33** | 1 | 0.0066 |
| 50 | **17-14-31** | 1 | 0.0066 |
| 51 | **20-13-28.2** | 1 | 0.0066 |
| 52 | **16-15-31** | 1 | 0.0066 |
| 53 | **18-14-33.2** | 1 | 0.0066 |
| 54 | **17-14-32** | 1 | 0.0066 |
| 55 | **19-16-30.2** | 1 | 0.0066 |
| 56 | **19-15-31.2** | 1 | 0.0066 |
| 57 | **16-13-34** | 1 | 0.0066 |
| 58 | **17-14-30** | 1 | 0.0066 |
| 59 | **19-15-32.2** | 1 | 0.0066 |
| 60 | **18-13-31.2** | 1 | 0.0066 |
| 61 | **18-12-28.2** | 1 | 0.0066 |
| 62 | **20-13-32** | 1 | 0.0066 |
| 63 | **19-14-33.2** | 1 | 0.0066 |
| 64 | **20-12-28.2** | 1 | 0.0066 |
| 65 | **18-14-32** | 1 | 0.0066 |
| 66 | **20-13-30.2** | 1 | 0.0066 |
| 67 | **20-13-31.2** | 1 | 0.0066 |
| 68 | **18-13-35** | 1 | 0.0066 |
| 69 | **19-13-28.2** | 1 | 0.0066 |

| **Supplementary Table S16. P-value for allele frequency distribution of 19 X-STR loci among the selected four ethnics data** | | | | | | |
| --- | --- | --- | --- | --- | --- | --- |
| **Locus** | **Han-Tibet** | **Han-Hui** | **Han- Uighur** | **Tibet-Uighur** | **Tibet-Hui** | **Uighur-Hui** |
| DXS10148 | 0.3580 | 0.1890 | 0.0002 | 0.0000 | 0.0010 | 0.0190 |
| DXS10135 | 0.0000 | 0.0560 | 0.0750 | 0.0000 | 0.0000 | 0.0140 |
| DXS8378 | 0.0030 | 0.9000 | 0.0200 | 0.0070 | 0.0480 | 0.0520 |
| DXS10159 | 0.2890 | 0.7570 | 0.0200 | 0.2600 | 0.3510 | 0.1310 |
| DXS10162 | 0.0210 | 0.6480 | 0.6980 | 0.0230 | 0.0010 | 0.1630 |
| DXS10164 | 0.0080 | 0.1980 | 0.5810 | 0.0710 | 0.2200 | 0.1000 |
| DXS7132 | 0.2200 | 0.4790 | 0.0010 | 0.0120 | 0.1270 | 0.0930 |
| DXS10079 | 0.1060 | 0.1000 | 0.0420 | 0.0130 | 0.1130 | 0.1380 |
| DXS10074 | 0.0010 | 0.3540 | 0.0000 | 0.0000 | 0.1490 | 0.0040 |
| DXS10075 | 0.3200 | 0.0890 | 0.0020 | 0.1960 | 0.5950 | 0.0660 |
| DXS6809 | 0.1450 | 0.5230 | 0.0000 | 0.1450 | 0.2330 | 0.0010 |
| DXS6789 | 0.0000 | 0.5290 | 0.0000 | 0.0000 | 0.0000 | 0.0000 |
| DXS7424 | 0.0000 | 0.4030 | 0.0020 | 0.0000 | 0.0000 | 0.0180 |
| DXS101 | 0.0290 | 0.4560 | 0.0000 | 0.0290 | 0.5980 | 0.0040 |
| DXS10103 | 0.2720 | 0.0850 | 0.0000 | 0.0000 | 0.0600 | 0.0860 |
| HPRTB | 0.0060 | 0.6920 | 0.0280 | 0.0000 | 0.0010 | 0.0050 |
| DXS10101 | 0.0060 | 0.0480 | 0.0000 | 0.0000 | 0.0000 | 0.0000 |
| DXS10134 | 0.0080 | 0.6850 | 0.0310 | 0.0210 | 0.0840 | 0.0300 |
| DXS7423 | 0.4500 | 0.7770 | 0.0000 | 0.0000 | 0.3260 | 0.0000 |

Han: Southern Han

| **Supplementary Table S17. P value for allele frequency distribution between the four selected population and previously published population data** | | | | | | | | |
| --- | --- | --- | --- | --- | --- | --- | --- | --- |
| Locus | Northern Han | Korean | Japanese | North German | Polish Tatars | North Italian | Spain | Ecuador Kichwa |
| The Southern Han population and previously published population data | | | | | | |  |  |
| DXS10148 |  |  |  |  |  |  |  |  |
| DXS10135 |  |  |  | 0.00000 |  |  |  |  |
| DXS8378 | 0.11503 | 0.00000 | 0.23491 | 0.00000 | 0.00000 | 0.00002 | 0.00000 | 0.00000 |
| DXS10159 |  |  |  |  |  |  |  |  |
| DXS10162 |  |  |  |  |  |  |  |  |
| DXS10164 |  |  |  |  |  |  |  |  |
| DXS7132 | 0.39891 | 0.07163 |  | 0.00028 | 0.03589 | 0.00000 | 0.00000 | 0.00030 |
| DXS10079 |  |  |  |  |  |  |  |  |
| DXS10074 |  |  |  | 0.00000 |  |  |  |  |
| DXS10075 |  |  |  |  |  |  |  |  |
| DXS6809 |  |  |  |  |  | 0.00000 | 0.00000 | 0.00000 |
| DXS6789 |  | 0.00000 | 0.00000 |  |  | 0.00126 | 0.00001 | 0.00441 |
| DXS7424 |  |  | 0.04120 |  |  | 0.00000 |  |  |
| DXS101 |  | 0.26469 | 0.57934 |  |  | 0.00000 |  |  |
| DXS10103 |  |  |  |  |  |  |  |  |
| HPRTB | 0.12093 | 0.17571 |  | 0.00056 | 0.00033 | 0.00004 |  |  |
| DXS10101 |  |  |  | 0.00000 |  |  |  |  |
| DXS10134 |  |  |  | 0.00000 |  |  |  |  |
| DXS7423 | 0.47681 | 0.32193 | 0.00484 | 0.00000 | 0.00000 | 0.00000 | 0.00000 | 0.00000 |
|  |  |  |  |  |  |  |  |  |
| **Supplementary Table S18. P value for allele frequency distribution between Tibet and previously published population data** | | | | |  |  |  |  |
| DXS10148 |  |  |  |  |  |  |  |  |
| DXS10135 |  |  |  | 0.00000 |  |  |  |  |
| DXS8378 | 0.00000 | 0.00000 | 0.00030 | 0.00000 | 0.00002 | 0.00058 | 0.00000 | 0.00000 |
| DXS10159 |  |  |  |  |  |  |  |  |
| DXS10162 |  |  |  |  |  |  |  |  |
| DXS10164 |  |  |  |  |  |  |  |  |
| DXS7132 | 0.00934 | 0.05437 |  | 0.01947 | 0.03745 | 0.00131 | 0.00012 | 0.00008 |
| DXS10079 |  |  |  |  |  |  |  |  |
| DXS10074 |  |  |  | 0.00000 |  |  |  |  |
| DXS10075 |  |  |  |  |  |  |  |  |
| DXS6809 |  |  |  |  |  | 0.00000 | 0.00000 | 0.00001 |
| DXS6789 |  | 0.00000 | 0.00000 |  |  | 0.00298 | 0.00006 | 0.00148 |
| DXS7424 |  |  | 0.00000 |  |  | 0.00000 |  |  |
| DXS101 |  | 0.03280 | 0.05043 |  |  | 0.00000 |  |  |
| DXS10103 |  |  |  |  |  |  |  |  |
| HPRTB | 0.00708 | 0.00039 |  | 0.00000 | 0.00000 | 0.00000 |  |  |
| DXS10101 |  |  |  | 0.00000 |  |  |  |  |
| DXS10134 |  |  |  | 0.00000 |  |  |  |  |
| DXS7423 | 0.05330 | 0.20863 | 0.00068 | 0.00000 | 0.00000 | 0.00000 | 0.00000 | 0.00000 |
|  |  |  |  |  |  |  |  |  |
| **Supplementary Table S19. P value for allele frequency distribution between Uighur and previously published population data** | | | | |  |  |  |  |
| DXS10148 |  |  |  |  |  |  |  |  |
| DXS10135 |  |  |  | 0.00000 |  |  |  |  |
| DXS8378 | 0.00001 | 0.00000 | 0.00002 | 0.05044 | 0.01307 | 0.31755 | 0.00001 | 0.00000 |
| DXS10159 |  |  |  |  |  |  |  |  |
| DXS10162 |  |  |  |  |  |  |  |  |
| DXS10164 |  |  |  |  |  |  |  |  |
| DXS7132 | 0.00846 | 0.36528 |  | 0.88729 | 0.00002 | 0.00516 | 0.52611 | 0.00004 |
| DXS10079 |  |  |  |  |  |  |  |  |
| DXS10074 |  |  |  | 0.00000 |  |  |  |  |
| DXS10075 |  |  |  |  |  |  |  |  |
| DXS6809 |  |  |  |  |  | 0.00000 | 0.00000 | 0.00000 |
| DXS6789 |  | 0.00000 | 0.00000 |  |  | 0.00781 | 0.00002 | 0.00032 |
| DXS7424 |  |  | 0.00000 |  |  | 0.00000 |  |  |
| DXS101 |  | 0.00000 | 0.00000 |  |  | 0.00319 |  |  |
| DXS10103 |  |  |  |  |  |  |  |  |
| HPRTB | 0.00009 | 0.00008 |  | 0.00466 | 0.00018 | 0.00177 |  |  |
| DXS10101 |  |  |  | 0.00000 |  |  |  |  |
| DXS10134 |  |  |  | 0.00000 |  |  |  |  |
| DXS7423 | 0.00000 | 0.00000 | 0.00000 | 0.01135 | 0.00003 | 0.00000 | 0.24306 | 0.00000 |
|  |  |  |  |  |  |  |  |  |
| **Supplementary Table S20. P value for allele frequency distribution between Hui population and previously published ten populations** | | | | | |  |  |  |
| DXS10148 |  |  |  |  |  |  |  |  |
| DXS10135 |  |  |  | 0.00000 |  |  |  |  |
| DXS8378 | 0.12180 | 0.00000 | 0.18735 | 0.00000 | 0.00000 | 0.00022 | 0.00000 | 0.00000 |
| DXS10159 |  |  |  |  |  |  |  |  |
| DXS10162 |  |  |  |  |  |  |  |  |
| DXS10164 |  |  |  |  |  |  |  |  |
| DXS7132 | 0.72205 | 0.33357 |  | 0.08248 | 0.02187 | 0.00004 | 0.00206 | 0.00015 |
| DXS10079 |  |  |  |  |  |  |  |  |
| DXS10074 |  |  |  | 0.00000 |  |  |  |  |
| DXS10075 |  |  |  |  |  |  |  |  |
| DXS6809 |  |  |  |  |  | 0.00000 | 0.00000 | 0.00000 |
| DXS6789 |  | 0.00000 | 0.00000 |  |  | 0.38503 | 0.00040 | 0.00618 |
| DXS7424 |  |  | 0.07078 |  |  | 0.00000 |  |  |
| DXS101 |  | 0.48902 | 0.38281 |  |  | 0.00000 |  |  |
| DXS10103 |  |  |  |  |  |  |  |  |
| HPRTB | 0.03281 | 0.10352 |  | 0.00388 | 0.00385 | 0.00007 |  |  |
| DXS10101 |  |  |  | 0.00000 |  |  |  |  |
| DXS10134 |  |  |  | 0.00000 |  |  |  |  |
| DXS7423 | 0.47322 | 0.35980 | 0.07877 | 0.00000 | 0.00000 | 0.00000 | 0.00000 | 0.00000 |

| **Supplementary Table S21. Maximum LOD score and female THETA in Southern Han 40 two-generation families** | | | | | | | | | | | | | | | | | | | |
| --- | --- | --- | --- | --- | --- | --- | --- | --- | --- | --- | --- | --- | --- | --- | --- | --- | --- | --- | --- |
| Female Theta\Max Lod Score | DXS10148 | DXS10135 | DXS8378 | DXS10159 | DXS10162 | DXS10164 | DXS7132 | DXS10079 | DXS10074 | DXS10075 | DXS6809 | DXS6789 | DXS7424 | DXS101 | DXS10103 | HPRTB | DXS10101 | DXS10134 | DXS7423 |
| DXS10148 |  | 0.029 | 0.001 | 0.319 | 0.293 | 0.356 | 0.340 | 0.329 | 0.369 | 0.297 | 0.360 | 0.329 | 0.301 | 0.300 | 0.281 | 0.213 | 0.272 | 0.343 | 0.305 |
| DXS10135 | 17.128 |  | 0.035 | 0.343 | 0.347 | 0.389 | 0.354 | 0.344 | 0.393 | 0.356 | 0.379 | 0.343 | 0.311 | 0.298 | 0.281 | 0.236 | 0.292 | 0.368 | 0.300 |
| DXS8378 | 19.238 | 13.396 |  | 0.333 | 0.306 | 0.359 | 0.350 | 0.352 | 0.389 | 0.321 | 0.368 | 0.323 | 0.291 | 0.302 | 0.294 | 0.208 | 0.286 | 0.333 | 0.311 |
| DXS10159 | 2.012 | 1.454 | 1.328 |  | 0.029 | 0.050 | 0.019 | 0.016 | 0.016 | 0.017 | 0.306 | 0.348 | 0.306 | 0.327 | 0.203 | 0.208 | 0.239 | 0.258 | 0.271 |
| DXS10162 | 2.868 | 1.483 | 2.071 | 16.551 |  | 0.022 | 0.041 | 0.029 | 0.016 | 0.033 | 0.225 | 0.236 | 0.227 | 0.266 | 0.196 | 0.203 | 0.234 | 0.261 | 0.288 |
| DXS10164 | 0.827 | 0.389 | 0.683 | 8.593 | 11.755 |  | 0.029 | 0.023 | 0.001 | 0.029 | 0.158 | 0.171 | 0.143 | 0.129 | 0.250 | 0.179 | 0.268 | 0.200 | 0.286 |
| DXS7132 | 1.058 | 0.900 | 0.794 | 13.507 | 11.122 | 8.564 |  | 0.001 | 0.001 | 0.001 | 0.279 | 0.347 | 0.262 | 0.275 | 0.239 | 0.206 | 0.245 | 0.277 | 0.250 |
| DXS10079 | 1.897 | 1.307 | 1.045 | 16.735 | 16.840 | 10.882 | 13.827 |  | 0.001 | 0.001 | 0.227 | 0.261 | 0.262 | 0.275 | 0.229 | 0.231 | 0.264 | 0.270 | 0.327 |
| DXS10074 | 0.977 | 0.606 | 0.584 | 17.029 | 17.029 | 10.220 | 12.625 | 16.833 |  | 0.001 | 0.293 | 0.306 | 0.276 | 0.309 | 0.222 | 0.227 | 0.275 | 0.241 | 0.313 |
| DXS10075 | 2.361 | 1.079 | 1.512 | 15.266 | 14.254 | 8.564 | 12.926 | 18.336 | 15.631 |  | 0.246 | 0.277 | 0.286 | 0.311 | 0.226 | 0.214 | 0.221 | 0.283 | 0.295 |
| DXS6809 | 1.294 | 0.851 | 0.867 | 2.071 | 4.920 | 4.241 | 1.888 | 4.506 | 2.223 | 3.359 |  | 0.058 | 0.092 | 0.073 | 0.280 | 0.322 | 0.289 | 0.303 | 0.269 |
| DXS6789 | 1.971 | 1.454 | 1.733 | 1.410 | 4.584 | 4.204 | 1.013 | 3.571 | 2.071 | 2.911 | 14.138 |  | 0.063 | 0.102 | 0.305 | 0.269 | 0.308 | 0.364 | 0.340 |
| DXS7424 | 2.572 | 1.930 | 2.154 | 2.071 | 4.506 | 5.163 | 2.154 | 3.118 | 2.623 | 2.307 | 10.877 | 12.768 |  | 0.001 | 0.280 | 0.241 | 0.296 | 0.359 | 0.339 |
| DXS101 | 2.144 | 2.074 | 1.857 | 1.455 | 3.177 | 4.155 | 1.824 | 2.336 | 1.786 | 1.430 | 10.331 | 9.336 | 15.932 |  | 0.191 | 0.265 | 0.258 | 0.314 | 0.293 |
| DXS10103 | 2.464 | 2.464 | 1.935 | 4.819 | 4.809 | 1.591 | 2.858 | 3.229 | 3.833 | 3.642 | 2.176 | 2.000 | 2.176 | 4.180 |  | 0.053 | 0.017 | 0.259 | 0.167 |
| HPRTB | 4.639 | 3.494 | 3.782 | 3.782 | 5.238 | 3.769 | 2.727 | 3.454 | 3.004 | 3.166 | 1.659 | 2.499 | 3.312 | 2.439 | 8.036 |  | 0.070 | 0.250 | 0.170 |
| DXS10101 | 3.810 | 2.799 | 2.596 | 4.401 | 4.995 | 1.987 | 2.905 | 3.629 | 3.135 | 4.887 | 3.019 | 2.571 | 2.649 | 3.288 | 15.559 | 10.869 |  | 0.261 | 0.164 |
| DXS10134 | 1.454 | 1.047 | 1.328 | 3.515 | 3.571 | 2.930 | 2.111 | 3.011 | 3.539 | 2.529 | 2.286 | 1.080 | 1.114 | 1.575 | 2.835 | 2.954 | 3.571 |  | 0.118 |
| DXS7423 | 2.000 | 1.787 | 1.430 | 2.274 | 2.375 | 1.442 | 2.045 | 1.381 | 1.502 | 1.647 | 2.499 | 1.132 | 1.279 | 1.578 | 4.425 | 4.836 | 5.912 | 7.330 |  |
| Max Lod Score > 3.0 means significant linkage | | | | |  |  |  |  |  |  |  |  |  |  |  |  |  |  |  |

| **Supplementary Table S22. allele frequency distribution between Southern Han and Guanzhong Han** | | |
| --- | --- | --- |
| Allele | P-value | X^2^ |
| DXS10148 | 0.2818 | 30.74 |
| DXS10135 | 0.0927 | 35.95 |
| DXS8378 | 0.4006 | 5.13 |
| DXS10159 | 0.0287 | 18.61 |
| DXS10162 | 0.5983 | 10.20 |
| DXS10164 | 0.0397 | 14.72 |
| DXS7132 | 0.5204 | 6.17 |
| DXS10079 | 0.4306 | 12.19 |
| DXS10074 | 0.4859 | 10.50 |
| DXS10075 | 0.4097 | 11.41 |
| DXS6809 | 0.0535 | 20.79 |
| DXS6789 | 0.0299 | 19.93 |
| DXS7424 | **0.0087** | 23.61 |
| DXS101 | 0.8068 | 8.54 |
| DXS10103 | 0.0669 | 16.00 |
| HPRTB | 0.3469 | 8.95 |
| DXS10101 | 0.6036 | 19.67 |
| DXS10134 | 0.0286 | 41.35 |
| DXS7423 | **0.0063** | 16.19 |
| P<0.01 indicated significant in bold | |  |

**Supplementary Figure S1. Manual of 19 X-STR System**

**AGCU** **X19 STR Kit**

AGCU X19 STR Kit is used to detect short tandem repeat (STR) loci, which utilizes five-dye fluorescent system (FAM for blue, HEX for green, TAMRA for yellow, ROX for red and SIZ for orange) and multiplex PCR techniques.

**1. Kit Components** (for 100 reactions)

| Pre-amplification Components | | | | |
| --- | --- | --- | --- | --- |
| Component | Size | | | Note |
| Reaction Mix | 1000μL×1 | | PCR buffer, MgCl_2_, dNTP mix | |
| X19 Primers | | 500μL×1 | 19 primer pairs | |
| A-Taq Polymerase | | 50μL×1 | 5U/μL | |
| Control DNA 9947A | | 20μL×1 | 0.5ng/μL9947A genomic DNA | |
| sdH_2_O | | 925μL×1 | Sterile deionized water | |
| Post-amplification Components | | | | |
| Component | | Size | Note | |
| X19 Allelic Ladder | | 25μL ×1 | Allelic ladders for 19 loci | |
| AGCU Marker SIZ-500 | | 125μL×1 | Internal DNA sizing standard | |

**Note**: You may need to vortex briefly and centrifuge shortly before using the reagents.

**2. PCR Reaction**


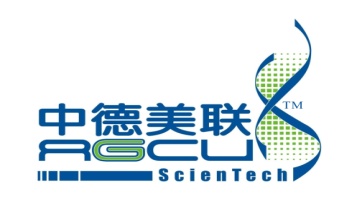


| Human genomic DNA | 0.5ng-2ng extracted genomic DNA |
| --- | --- |
| Reaction Mix | 10.0μL |
| X19 Primers | 5.0μL |
| A-Taq Polymerase | 0.5μL |
| sdH_2_O | to 25.0μL |

Thermal Cycling Parameters:

| Initial Incubation  Step | Hold | 95ºC 2min |
| --- | --- | --- |
| Cycle 1 For 10 cycles | Denature | 94ºC 30sec |
|  | Anneal | 60ºC 1min |
|  | Extend | 65ºC 1min |
| Cycle 2 For 20 cycles | Denature | 94ºC 30sec |
|  | Anneal | 59ºC 1min |
|  | Extend | 72ºC 1min |
| Final Extension | Hold | 60ºC 30min |
| Final Step | Hold | 4ºC forever |

Note: Amplification parameters and detection instruments may vary. You may need to optimize protocols including cycle numbers and annealing temperature for each laboratory instrument.

3. Electrophoresis

Prepare a loading cocktail by combining 0.5μL of AGCU Marker SIZ-500, 9.5μL of Hi-Di™ formamide and 1μL of amplified sample (or 0.5-1μl of X19 allelic ladder mix). Heat samples to 95°C for 3 minutes to denature, then immediately chill on crushed ice or in an ice-water bath for 3 minutes. And then start electrophoresis.

**Genotyping Results of 9947A** (0.5ng/25μL)


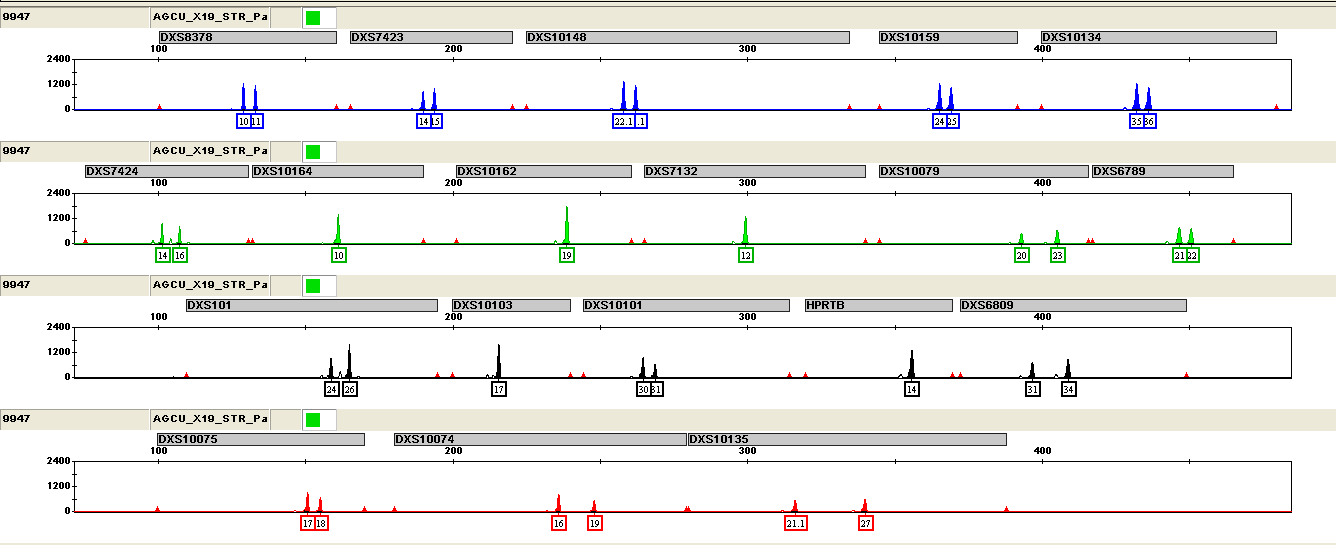

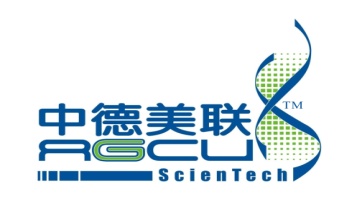


**Information of 19 X-STR loci**

| Locus | Label | X19 Allelic Ladder | 9947A  Genotype |
| --- | --- | --- | --- |
| DXS8378 | FAM | 8,9,10,11,12,13 | 10/11 |
| DXS7423 | FAM | 13,14,15,16,17 | 14/15 |
| DXS10148 | FAM | 17,18,19,20,21,22.1,23.1,24.1,25.1,26.1,27.1,  28.1,29.1,30.1,31.1,32.1,33.1 | 22.1/23.1 |
| DXS10159 | FAM | 21,22,23,24,25,26,27,28 | 24/25 |
| DXS10134 | FAM | 31,32,33,34,35,36,37,38,39,40,41,42.3,43.3 | 35/36 |
| DXS7424 | HEX | 9,11,12,13,14,15,16,17,18,19,20 | 14/16 |
| DXS10164 | HEX | 8,9,10,11,12,13,14 | 10/10 |
| DXS10162 | HEX | 14,15,15.2,16,17,17.2,18,19,20,21,22 | 19/19 |
| DXS7132 | HEX | 11,12,13,14,15,16,17,18 | 12/12 |
| DXS10079 | HEX | 15,16,17,18,19,20,21,22,23 | 20/23 |
| DXS6789 | HEX | 15,16,17,18,19,20,21,22,23 | 21/22 |
| DXS101 | TAMRA | 18,19,20,21,22,23,24,25,26,27,28,29,30,31,32,33 | 24/26 |
| DXS10103 | TAMRA | 15,16,17,18,19,20,21 | 17/17 |
| DXS10101 | TAMRA | 26.2,27,28,28.2,29,29.2,30,30.2,31,31.2,32,  32.2,33,34,35 | 30/31 |
| HPRTB | TAMRA | 8,9,10,11,12,13,14,15,16 | 14/14 |
| DXS6809 | TAMRA | 25.2,26.2,29,30,31,32,33,34,35 | 31/34 |
| DXS10075 | ROX | 12,13,14,14.2,15,16,16.2,17,17.2,18,18.2,19 | 17/18 |
| DXS10074 | ROX | 12,13,14,15,16,17,18,19,20,21,22 | 16/19 |
| DXS10135 | ROX | 14,16,18,19,20,21,22,23,24,25,26,27,28,29,30,  31,32,33,34,35,36 | 21.1/27 |
